# Mitochondrial Genomics of Six Cacao Pathogens From the Basidiomycete Family *Marasmiaceae*

**DOI:** 10.3389/fmicb.2021.752094

**Published:** 2021-10-28

**Authors:** Shahin S. Ali, Ishmael Amoako-Attah, Jonathan Shao, Eric Kumi-Asare, Lyndel W. Meinhardt, Bryan A. Bailey

**Affiliations:** ^1^Sustainable Perennial Crops Laboratory, U. S. Department of Agriculture (USDA)/Agricultural Research Service (ARS), Beltsville Agricultural Research Center-West, Beltsville, MD, United States; ^2^Department of Viticulture and Enology, University of California, Davis, Davis, CA, United States; ^3^Cocoa Research Institute of Ghana, Akim Tafo, Ghana; ^4^U. S. Department of Agriculture (USDA)/Agricultural Research Service (ARS), Beltsville, MD, United States

**Keywords:** mitochondrial genome (mitogenome), basidiomycete, *Marasmiaceae*, thread blight disease, cacao (*Theobroma cacao*)

## Abstract

Thread blight disease has recently been described as an emerging disease on cacao (*Theobroma cacao*) in Ghana. In Ghana, thread blight disease is caused by multiple species of the *Marasmiaceae* family: *Marasmius tenuissimus, M. crinis-equi*, *M. palmivorus*, and *Marasmiellus scandens*. Interestingly, two additional members of the *Marasmiaceae*; *Moniliophthora roreri* (frosty pod rot) and *Moniliophthora perniciosa* (witches’ broom disease), are major pathogens of cacao in the Western hemisphere. It is important to accurately characterize the genetic relationships among these economically important species in support of their disease management. We used data from Illumina NGS-based genome sequencing efforts to study the mitochondrial genomes (mitogenomes) of the four cacao thread blight associated pathogens from Ghana and compared them with published mitogenomes of *Mon. roreri* and *Mon. perniciosa*. There is a remarkable interspecies variation in mitogenome size within the six cacao-associated *Marasmiaceae* species, ranging from 43,121 to 109,103 bp. The differences in genome lengths are primarily due to the number and lengths of introns, differences in intergenic space, and differences in the size and numbers of unidentified ORFs (uORF). Among seven *M. tenuissimus* mitogenomes sequenced, there is variation in size and sequence pointing to divergent evolution patterns within the species. The intronic regions show a high degree of sequence variation compared to the conserved sequences of the 14 core genes. The intronic ORFs identified, regardless of species, encode GIY-YIG or LAGLIDADG domain-containing homing endonuclease genes. Phylogenetic relationships using the 14 core proteins largely mimic the phylogenetic relationships observed in gene order patterns, grouping *M. tenuissimus* with *M. crinis-equi*, and *M. palmivorus* with *Mon. roreri* and *Mon. perniciosa*, leaving *Mar. scandens* as an outlier. The results from this study provide evidence of independent expansion/contraction events and sequence diversification in each species and establish a foundation for further exploration of the evolutionary trajectory of the fungi in *Marasmiaceae* family.

## Introduction

There are more than 1,000 species of fungi in the family *Marasmiaceae*, which includes the genus *Marasmius* Fr. (1836: 339) (*Agaricales, Basidiomycota*). Although most are saprobes, the family does include plant pathogens. Examples include *Marasmiellus scandens* on cacao and other tropical trees (Massee, 1910), *Marasmius crinis-equi* on cacao and the tea tree (Su et al., 2011), *M. puerariae* on kudzu vine (Kirschner et al., 2013), *M. palmivorus* on cacao and oil and coconut palms (Desjardin and Perry, 2017) and *M. graminum* on rice (Gaire et al., 2020). In addition, two devastating cacao diseases, frosty pod rot and witches’ broom disease which occur in the Western hemisphere, are caused by the *Marasmiaceae* species *Moniliophthora roreri* and *Moniliophthora perniciosa*, respectively (Evans et al., 2003).

Thread blight disease (TBD) is an emerging threat to cacao production in Ghana (Amoako-Attah et al., 2016, 2020). It is caused by canopy-dwelling basidiomycetes and mostly seen as a network of mycelial rhizomorphs, often called “threads,” that grow along twigs and branches covering leaves creating distinctive leaf blight symptoms. Historically, based on morphological methods, TBD in cacao has been attributed to the causal agent *Mar. scandens* (syn: *M. byssicolor*) in Ghana and in other cacao growing areas of the world (Opoku et al., 2007; Amoako-Attah et al., 2016). However, recent morphological and molecular analyses identified four different fungal species, of the *Marasmiaceae*, causing TBD- like symptoms on cacao in Ghana (Amoako-Attah et al., 2020). The four species were *M. crinis-equi* (abundant thin, black, “horse hair”-type rhizomorphs), *M. tenuissimus* (scattered brown or whitish to brownish-white rhizomorphs), *M. palmivorus* (aggregates of shiny- or silky-white hyphae), and *Mar. scandens* (faint-cream or dull-white hyphae). Among the four species, *M. tenuissimus* was the most frequently isolated TBD-associated fungus in Ghana (Amoako-Attah et al., 2020). When considered with the *Moniliophthora* spp. that also cause disease on cacao, these six species within the *Marasmiaceae* create a fertile ground for dissection of genetic diversity in association with varied disease processes in a single crop.

Mitochondria play important roles in eukaryotic cells, mainly with respect to respiratory metabolism and energy supply (Kobayashi et al., 1996). Production of reactive oxygen species during ATP synthesis within the mitochondria makes the mitochondrial DNA more susceptible to damage and mutations, compared to nuclear DNA (Richter, 1992). At the same time, the mitochondrial genome is subject to all the mechanisms responsible for maintaining nuclear genome integrity (Kaniak-Golik and Skoneczna, 2015). Recent findings suggest that the mutation rate of mitochondrial DNA can be significantly lower in plants and fungi than in animals, with some exceptions (Torriani et al., 2014; Sandor et al., 2018). Fungal mitochondrial genomes (mitogenomes) are typically small and present in multiple copies in each cell with a highly compact gene organization (Franco et al., 2017). Mitogenomes usually harbor 14 core-genes encoding proteins involved in electron transport and oxidative phosphorylation, as well as untranslated genes of the small and large ribosomal RNA (rRNA) subunits and a set of transfer RNA (tRNA) genes (Bullerwell and Lang, 2005). Fungal mitogenomes sometimes also carry genes encoding the mitochondrial ribosomal protein S3 (*rps3*) and the RNA subunit of the mitochondrial RNase P (Franco et al., 2017). Beside these genes, fungal mitogenomes are also characterized by their variable number of group I and group II introns that may carry open reading frames with motifs of homing endonuclease genes (HEGs) (Bullerwell and Lang, 2005; Lang et al., 2007). HEGs are genetic mobile elements that encode site-specific DNA endonucleases and promote their own propagation (Burt and Koufopanou, 2004; Edgell, 2009) resulting in the insertion, deletion or mutation of DNA sequences (Stoddard, 2011). Such, mobile introns like HEGs represent one of the major sources of variation within fungal mitogenomes (Wu and Hao, 2014; Repar and Warnecke, 2017). Fungal mitogenomes often differ in gene order and composition, pseudogene content and length of intergenic regions due to rearrangements caused by recombination. The presence of double-stranded RNA elements and self-splicing introns can also add to fungal mitogenome variability (Kulik et al., 2020). Variations in mitogenomes can provide vital clues into the evolution, population genetics, and biology of the organisms involved and are a rich source of novel genotyping markers due to the presence of these mobile introns.

Unlike plants and animals, fungi exhibit a diversity of mitochondrial DNA inheritance patterns from strictly uniparental to biparental, or a mixture of both, as well as recombinant mitochondrial DNA genotypes (Wilson and Xu, 2012; Xu and Wang, 2015). Uniparental mitochondrial inheritance is very common in filaments basidiomycete (Xu and Li, 2015). Moreover, *Mon. roreri* propagates clonally (Ali et al., 2015; Díaz-Valderrama and Aime, 2016), whereas *Mon. perniciosa* is primarily homothallic (Kües and Navarro-González, 2010), and thus is expected to have primarily uniparental mitochondrial DNA inheritance. The mitochondrial DNA inheritance patterns of other *Marasmiaceae* need further confirmation.

With the emergence of easier and more affordable next generation sequencing, access to whole genome sequences has increased dramatically, along with the number of completely sequenced mitogenomes. But within the *Marasmiaceae* family, only two complete mitogenomes from *Mon. roreri* and *Mon. perniciosa* have been reported (Formighieri et al., 2008; Costa et al., 2012), representing a gap in terms of understanding the evolutionary and biological aspects of this large group of fungi. As emerging pathogens of cacao and other tropical crops, it is important to understand the genomics of these economically important *Marasmiaceae* species. Therefore, we sequenced and assembled the complete mitogenomes of *M. crinis-equi*, *M. tenuissimus*, *M. palmivorus*, and *Mar. scandens*. As *M. tenuissimus* was the major causal agent TBD in Ghana and reported to have different morphotypes (Amoako-Attah et al., 2020), we also sequenced and assembled the complete mitogenomes of six additional *M. tenuissimus* isolates and performed a comparative mitogenome analysis along with *Mon. roreri* and *Mon. perniciosa*.

## Materials and Methods

### Fungal Isolates

Fungal isolates were isolated from TBD samples collected across Ghana as reported previously (Amoako-Attah et al., 2020). Based on that previous study (Amoako-Attah et al., 2020), one strain each from *M. crinis-equi*, *M. palmivorus* and *Mar. scandens*, and seven isolates from *M. tenuissimus* (Table 1) were chosen for whole genome Illumina sequencing and their complete mitogenomes were assembled for this study. Fungal biomass generation and DNA extraction was performed as reported previously (Amoako-Attah et al., 2020).

**TABLE 1:** Mitogenome statistics of four cacao thread blight associated pathogens from Ghana and the Western hemisphere frosty pod rot (*Moniliophthora roreri*) and witches’ broom (*Mon. perniciosa*) pathogens of cacao, all basidiomycetes within the *Marasmiaceae* family.

Species*	**Isolate/GenBank accession**	**Length of mitochondrial genome (bp)**	**% GC**	**No. of tRNAs**	**No. of protein-coding genes**	**Genes with introns**	**No. of introns**	**No. of accessory genes/ORFs**	**No of intronic ORFs**	**Core genes (bp)**	**tRNA (bp)**	**Accessory genes (bp)**	**Introns (bp)**	**Intronic ORFs (bp)**	**Intergenic regions (bp)**	**% of Intergenic regions**
*Mar. scandens*	GHA19/MZ615350	86,201	32.04	25	29	4	9	15	8	30,349	1,850	14,478	11,413	8,293	36,404	42.23
*M. crinis-equi*	GHA76/MZ615351	63,005	28.47	24	22	2	7	8	6	27,613	1,776	9,517	8,838	5,760	21,021	33.36
*M. palmivorus*	GHA12/MZ615352	79,017	26.58	26	34	2	7	20	6	29745	1,930	15,014	8,380	5,631	29,579	37.43
*M. tenuissimus*	GHA37/MZ615348	44,399	26.97	25	18	1	1	4	1	19,321	1,848	4,697	1,054	956	18,435	41.52
*M. tenuissimus*	GHA63/MZ615349	51,210	27.21	25	21	1	3	7	3	23,274	1,848	9,008	3,323	2,853	16,610	32.44
*M. tenuissimus*	GHA64/MZ615347	43,121	26.66	25	19	1	1	5	1	19,374	1,848	4,810	1,054	956	16,991	39.40
*M. tenuissimus*	GHA74/MZ615346	44,859	27.01	25	17	1	1	3	1	18,957	1,848	4,935	1,054	956	19,021	42.40
*M. tenuissimus*	GHA79/MZ615345	48,952	26.82	26	21	1	3	7	3	21,833	1,919	7,985	3,255	2,835	16,795	34.31
*M. tenuissimus*	MS2/MZ615344	44,524	27.02	25	18	1	1	4	1	19,638	1,848	4,697	1,054	956	18,243	40.97
*M. tenuissimus*	GHA07/MZ615343	44,461	27.04	25	17	1	1	4	1	17,863	1,848	4,697	1,054	956	19,955	44.88
*Mon. roreri*	HQ259115.1	93,722	27.6	27	52	4	13	38	14	36,952	2,013	28,643	16,979	13,228	22,363	23.86
*Mon. perniciosa*	AY376688.1	109,103	31.9	26	64	6	13	50	10	38,345	1,934	32,820	15,482	9,125	29,647	27.17

*Mar.: Marasmiellus, M.: Marasmius, Mon.: Moniliophthora.

### Sequencing and Mitogenome Assembly

Genomic DNA of the above mentioned 10 isolates were sequenced using Illumina X-ten paired-end short-read technology (library preparation and sequencing done by Beijing Genome Institute, Shenzhen, China) as reported previously (Ali et al., 2020). The short reads were assembled using ‘‘SOAPdenovo’’ (^1^ version: 2.01) as described previously (Ali et al., 2017). The mitogenomes were identified from the assembled contigs based on the average GC content, size and depth of coverage of the contigs. As the fungal mitogenomes are normally AT-rich (Hausner, 2003), we targeted the contigs with < 35% GC content, high coverage and >1,000 bp long, and these were BLASTn (Altschul et al., 1990) against the *Mon. roreri* mitogenome (Costa et al., 2012). Only one contig per isolate showed similarity on the nucleotide level to the *Mon. roreri* mitogenome and showed the presence of common mitochondrial genes. To confirm the circularity of the mitogenomes, contigs were searched for sequence repeats on each end and trimmed leaving only the unique sequence. The mitogenomes from all the 10 isolates satisfied that criteria.

### Annotation of the Mitogenome

Mitochondrial gene annotation of the 10 isolates and the publicly available *Mon. roreri* (GenBank no. HQ259115.1) and *Mon. perniciosa* (GenBank no. AY376688.1) were performed with MFannot (Valach et al., 2014) using the NCBI translation code 4 (The Mold, Protozoan, and Coelenterate Mitochondrial Code). The tRNAs and introns were re-confirmed by RNAweasel^2^. To further confirm genes boundaries as well as intron-exon boundaries, MFannot predictions were again checked individually by aligning against their orthologous in closely related fungal species. A physical map of the mitogenome was created with OrganellarGenome-DRAW (OGDRAW) v 1.2 (Lohse et al., 2007). Functional annotation of the predicted open reading frames (ORFs) was complemented with Blast2GO Basic (Conesa et al., 2005). The mitogenomes were deposited at NCBI GenBank under the accession number MZ615343- MZ615352.

### Phylogenetic Inference

To evaluate the application of mitogenomes for fungal phylogeny, a phylogenetic tree was constructed using Amino acid sequences of the 14 conserved mitochondrial proteins of these species, including three ATP synthase subunits (*atp6*, *atp8*, and *atp9*), four Cytochrome c oxidase subunits (*cox1*, *cox2*, *cox3*, and *cob*), and seven NADH dehydrogenase subunits (*nad1*, *nad2*, *nad3*, *nad4*, *nad4L*, *nad5*, and *nad6*). Accessions of completely sequenced mitogenomes of four species in *Marasmiaceae* family and five species from other families of the order Agaricales were retrieved from NCBI Organelle Genome Resources website^3^. In addition, the mitogenomes of *Rhizoctonia solani* was used as outgroups. Protein sequences were combined and aligned using ClustalW2 tool (Larkin et al., 2007) under default settings and a phylogenetic tree was reconstructed using the Maximum Likelihood method based on the JTT matrix-based model using MEGA v. 6 (Tamura et al., 2013). The best fit amino acid substitution model with lowest BIC score was determined using MEGA v. 6. Bootstrap values were computed with 1000 resampling iterations using an approximate likelihood ratio test. Similarly, phylogenetic trees were also constructed using Amino acid sequences of the *rps3* gene, cox1 introns and the internal and external ORF’s of *Marasmiaceae* species listed in Table 1. The best fit model used, as determined using MEGA v. 6, for these phylogenetic trees were as follows: rps3 of three *M. tenuissimus* isolates with the other five species- WAG; rps3 of seven isolates of *M. tenuissimus*- JTT; *cox1* introns of two *M. tenuissimus* isolates with the other five species- GTR; *cox1* introns of seven isolates of *M. tenuissimus*- T92; and the internal and external ORF’s of all the six *Marasmiaceae* species- WAG.

### Mitogenome Synteny and Structural Comparisons

The complete mitogenomes of *Marasmiaceae* species listed in Table 1 were used for reciprocal BLASTn searches to identify regions of similarity, insertions, and rearrangements. First, to identify major rearrangements, structural comparisons between the three types of mitogenomes based on gene order (*M. tenuissimus*, *Mar. scandens*, and *M. palmivorus*) were generated with Circoletto (Darzentas, 2010), combining BLASTn (threshold of 1e ^––10^) searches with the Circos output. Second, to identify regions of similarity and insertions, complete mitogenome alignments were carried out using the mVISTA program^4^. Default parameters were utilized to align the genomes in Shuffle-LAGAN mode and a sequence conservation profile was visualized in an mVISTA plot (Frazer et al., 2004).

### GC-Plots

To show the distribution of the GC content along the mitogenomes, GC-plots were created using DNAplotter (Release 18.1.0) with a window size of 10,000 bp, and step size of 200 bp. The pink represents below average and the green represents above average GC content for each mitogenomes.

### Tandem Repeats (TRs)

Tandem repeats were identified using the Tandem Repeats Finder (Benson, 1999) under default parameters.

## Results and Discussion

### Overview of the Mitogenomes

**FIGURE 1:**
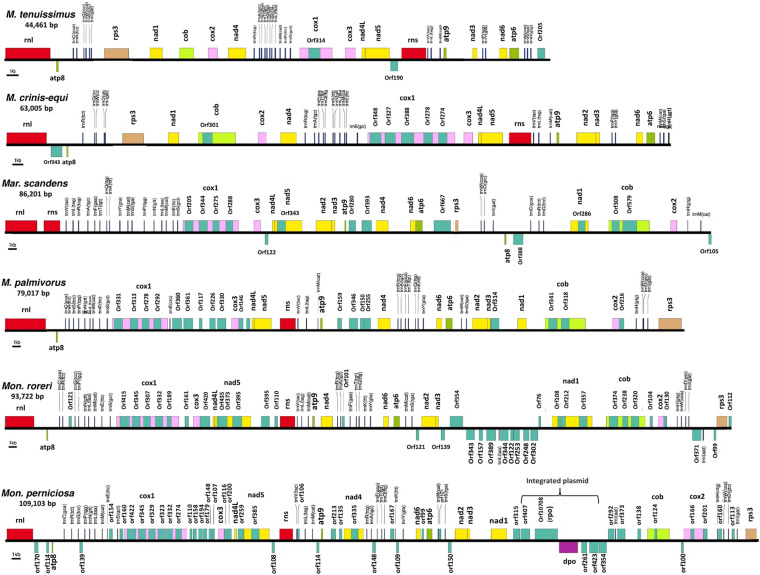
Conservation of gene order and interspecies variation in the mitogenome structure of four cacao thread blight associated pathogens from Ghana and the Western hemisphere frosty pod rot and witches’ broom pathogens of cacao, all basidiomycetes within the *Marasmiaceae* family. Whole mitochondrial genome sequence involved are *Marasmius tenuissimus* (GHA07), *Marasmiellus scandens* (GHA19), *Marasmius crinis-equi* (GHA76); *Marasmius palmivorus* (GHA12), *Moniliophthora roreri* (Genbank no. HQ259115.1), *Moniliophthora perniciosa* (Genbank no. AY376688.1). Mitochondrial gene annotation was performed with MFannot using the NCBI translation code 4, and the physical map of the mitogenomes were created with OrganellarGenome-DRAW (OGDRAW) v 1.2.

The mitogenomes of *M. crinis-equi* (strain: GHA76), *M. palmivorus* (strain: GHA12), *Mar. scandens* (strain: GHA19), and seven isolates of *M. tenuissimus* were sequenced, assembled, and annotated in this study (Table 1 and Supplementary Excel File 1). The *Mon. roreri* (GenBank no. HQ259115.1) and *Mon. perniciosa* (GenBank no. AY376688.1) mitogenomes were originally annotated manually using BLAST searches and ORF finder software (Formighieri et al., 2008; Costa et al., 2012). For comparative genomics, it is important to use the same annotation technique. Therefore, the *Mon. roreri* and *Mon. perniciosa* mitogenomes were re-annotated using the MFannot tool (Valach et al., 2014). Both the current and previous methods resulted in the same number and orientation of the primary mitochondrial genes and similar numbers of intronic and accessory ORFs (Supplementary Figure 1). Mitogenomes are known for their wide range of sizes despite their seemingly similar functional gene composition, even among closely related species (Zhang et al., 2020). There is a remarkable interspecies variation in mitogenome size within the six cacao-associated *Marasmiaceae* species so far sequenced, ranging from 43,121 to 51,210 bp for *M. tenuissimus*, to 63,005 bp (*M. crinis-equi*), 79,017 bp (*M. palmivorus*), and 86,201 bp (*Mar. scandens*), as compared to 93,722 bp for *Mon. roreri* and 109,103 bp for *Mon. perniciosa* (Table 1). A portion of *Mon. perniciosa’s* expanded genome size can be attributed to the stable insertion of a linear plasmid (Formighieri et al., 2008) between *nad1* and *cob* in association with other rearrangements (Figure 1).

### Primary Mitochondrial Gene Composition and Order

**FIGURE 2:**
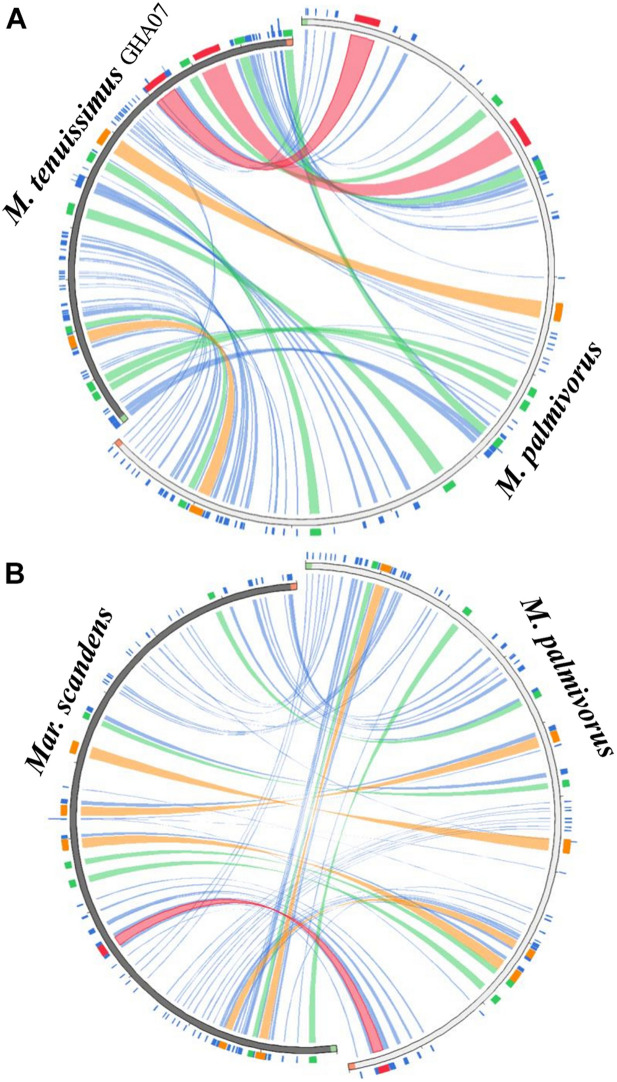
Circos plot mapping the block rearrangements, synteny and identity level of **(A)** *Marasmius tenuissimus* and **(B)** *Marasmiellus scandens* against *Marasmius palmivorus* mitogenomes. Green and red block in the beginning and end of the sequences indicate the sequence orientation. Colors blocks and ribbons represent the local alignments produced by BLAST the score/max bits core ratio, with blue ≤ 0.25, green ≤ 0.50, orange ≤ 0.75, and red > 0.75 being the best quartile. The figure was produced using Circoletto (Darzentas, 2010).

**FIGURE 3:**
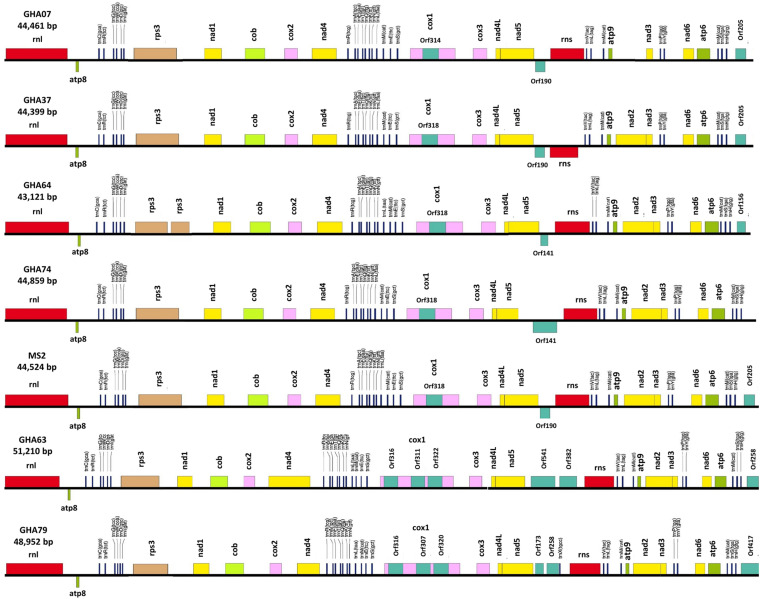
Conservation of gene order and Intraspecies variation in the mitogenome structure of seven isolates of the cacao thread blight associated pathogen *Marasmius tenuissimus.* Mitochondrial gene annotation was performed with MFannot using the NCBI translation code 4, and the physical map of the mitogenomes were created with OrganellarGenome-DRAW (OGDRAW) v 1.2.

All the mitogenome sequences (*M. crinis-equi*, *M. palmivorus*, *Mar. scandens*, seven isolates of *M. tenuissimus*, *Mon. roreri*, and *Mon. perniciosa*) carry the 14 core genes (Bullerwell and Lang, 2005) involved in oxidative phosphorylation, ATP synthesis and mitochondrial protein synthesis (Figure 1 and Supplementary Excel File 1). In addition, these genomes carry genes encoding the small and large subunits of rRNA. All the rRNA and core genes were in the same orientation, except for *atp8*. Though it is not common in fungi for *atp8* be in the opposite orientation, it seems to be a common phenomenon in the *Marasmiaceae.* Beside the above mentioned six *Marasmiaceae* species, *Lentinula edodes and Omphalotus japonicus* maintained the same orientation (Supplementary Figure 2). But it is not a common phenomenon for the order *Agaricales* or class *Agaricomycetes* (Supplementary Figure 2). All the genomes also carry a complete set of tRNAs and the *rps3* gene (Supplementary Excel File 1). The tRNAs are generally distributed in small blocks around the genomes, the exception being *Mar. scandens* which has 17 of 25 tRNA copies located in a single block between the rns and *cox1* genes (Figure 1). Further analysis comparing the mitogenome structure of *Mar. scandens* with that of *M. tenuissimus* and *M. palmivorus* indicates that the large tRNA block of *Mar. scandens* has resulted from recombination of at least four small tRNA blocks (Supplementary Figure 3), suggesting a recombination hotspot in the *Mar. scandens* mitogenome. The gene order for the core genes within the mitogenomes for the six species varied and show distinct relationships depending on the species compared. *M. palmivorus* has a conserved gene order aligning with *Mon. roreri* and *Mon. perniciosa* for both core genes and tRNAs (Figure 1 and Supplementary Figure 4). Similarly, *M. crinis-equi* and *M. tenuissimus* have a conserved gene order with each other but not with the other species being studied (Figure 1 and Supplementary Figure 5). When compared to the other species being studied, the *Mar. scandens* mitogenome sequence is unique (Figure 1). Comparing these three patterns of gene order, multiple gene block rearrangements are evident (Figure 2). All the seven *M. tenuissimus* isolates showed a conserved gene order despite differences in their mitogenome sizes and ORF composition (Figure 3 and Supplementary Figure 6). Though the mechanisms of gene rearrangement in fungal mitogenomes are not fully understood, these gene arrangements provide a large amount of information for understanding the evolutionary status of species (Wu et al., 2021).

### Phylogenetic Analysis

**FIGURE 4:**
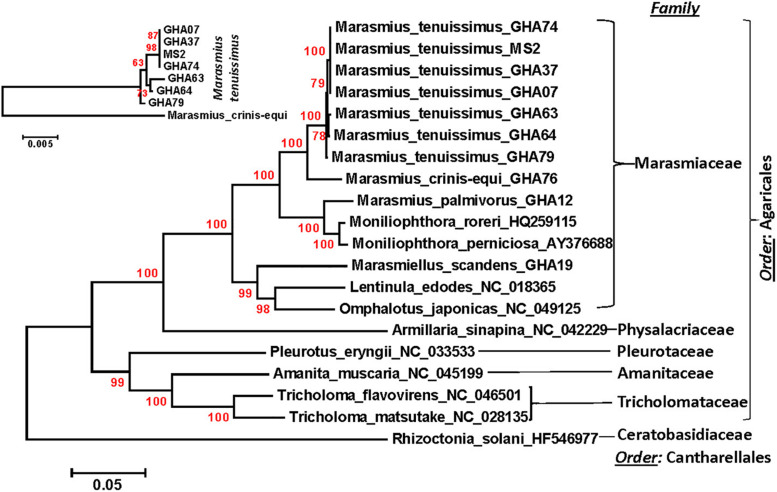
Molecular phylogenetic analysis of four cacao thread blight associated pathogens from Ghana (*M. tenuissimus*, *M. palmivorus*, *M. crinis-equi*, *Mar. scandens*) and the Western hemisphere frosty pod rot (*Mon. roreri*) and witches’ broom (*Mon. perniciosa*) pathogens of cacao along with some other members of *Marasmiaceae* and *Agaricales*. The *Rhizoctonia solani* was used as an outgroup. The analysis was based on the amino-acid sequences of 14 conserved mitochondrial proteins with 3,154 distinct alignment positions and 1,000 rapid bootstrap inferences. Sequences were combined and aligned using ClustalW2 tool under default setting and the phylogenetic tree was reconstructed using the Maximum Likelihood method. The tree is drawn to scale, with branch lengths measured in the number of substitutions per site. Analyses were conducted in MEGA6 (Tamura et al., 2013). The analysis was re-run with seven *M. tenuissimus* and *M. crinis-equi* as an outgroup.

A phylogenetic analysis was carried out incorporating the 14 concatenated core proteins for each of the 10 mitogenomes sequenced in this study along with additional *Marasmiaceae* and Agaricales mitogenomes obtained from the public domain (Figure 4). The resulting phylogenetic tree separated each species within the *Marasmiaceae* family with bootstrap support of more than 98%. The tree topology shows three distinct clades (each with internal nodes of 99–100% bootstrap support) with *M. palmivorus, Mon. roreri*, and *Mon. perniciosa* forming one clade, *M. crinis-equi* and *M. tenuissimus* forming another clade and *Mar. scandens* grouping with L. edodes and *O. japonicus* to form a third clade. Notably, similar grouping was also observed based on the conservation of gene order for the first two clades. But there was no conserved gene order between *Mar. scandens*, *L. edodes* and *O. japonicus* (Figure 1 and Supplementary Figure 2). The presence a of large tRNA block in both *Mar. scandens* and *O. japonicus* further supports the presence of a recombination hotspot possibly contributing to the non-conserved gene order in this clade. These phylogenetic findings were consistent with earlier phylogenetic observations based on ITS sequence of a large group of *Marasmiaceae* fungi from around the world (Amoako-Attah et al., 2020). In addition to clearly showing interspecies variation, the phylogenetic tree also showed some intraspecies variation within the seven isolates of *M. tenuissimus* with bootstrap support of more than 78% (Figure 4). Four of the five *M. tenuissimus* isolates, each carrying a single intron in the *cox1* gene (Figure 3), showed 100% sequence similarity (Figure 4). *M. tenuissimus* isolate GHA64, also carrying a single *cox1* intron and having the smallest genome, was separated from the four isolates with bootstrap support of 79% (Figure 4) and sequence similarity of 99.75% suggesting this variation was not critically linked to variation in *cox1* intron composition. *M. tenuissimus* isolates GHA79 and GHA63, both carrying three *cox1* introns, were not clonal (Figure 4). Amoako-Attah et al. (2020) also reported different morphotypes within *M. tenuissimus* isolates. GHA79 was reported as morphotype B and GHA63 was reported as morphotype C, further indicating the isolates are not clonal. Whether these three different phylogenetic groups within the *M. tenuissimus* are different cryptic species, need further investigation. The whole genome sequence analysis of these isolates which is ongoing in our lab should shed more light on this subject. The rRNA ITS sequence is the only molecular marker so far used in *Marasmiaceae* phylogeny (Koch et al., 2018; Amoako-Attah et al., 2020). The mitogenome sequence provide more genetic information than the ITS sequence alone, and is a better choice for analyzing the origin and phylogeny of this lesser known family of *Basidiomycetes.*

### Mitogenome Size Variation and Sources of Genome Expansion

Despite similarities in primary gene compliments and, in specific comparisons, gene order associations, the genome lengths vary significantly both between closely related species and, for *M. tenuissimus*, within species. These differences in genome lengths are primarily due to the number and lengths of introns, differences in intergenic space, and differences in the size and numbers of unidentified ORFs (uORF). The numbers of introns found in the 12 genomes compared ranged from 1 to 13 with total lengths varying from 1,054 to 16,979 bp. *Mon. roreri* has four protein coding genes (*cox1*, *nad5*, *nad1*, and *cob*) with introns. *Mon. perniciosa* has 6 (*cox1*, *nad5*, *nad4*, *nad1*, *cob*, and *cox2*), *Mar. scandens*, *M. crinis-equi* and *M. palmivorus* have 2 (*cox1*, *cob*), and *M. tenuissimus* has 1 (*cox1*) protein coding gene(s) with introns. It is typical in fungi that *cox1* carries the most intronic sequences and *cob* carries the second most for a specific species (Megarioti and Kouvelis, 2020). *Mon. roreri* has 5 *cox1* introns, *Mon. perniciosa* 6 *cox1* introns, *Mar. scandens* 4 *cox1* introns, *M. crinis-equi* 5 *cox1* introns and *M. palmivorus* 4 *cox1* introns (Figure 1). *M. tenuissimus* isolates GHA63 and GHA79 carry 3 introns within the *cox1* gene while the remaining isolates carry only one intron within the *cox1* gene (Figure 3). Another discrepancy observed is the *nad4* gene of *M. tenuissimus* isolates GHA63 which is almost double in size compared to the other isolates and carries no intron (Figure 3 and Supplementary Excel File 1). The intronic regions show a high degree of sequence variation compared to the conserved sequences of the 14 core genes (Supplementary Figures 4, 5). Variation in intron numbers and length typically contributes to the size variation of the fungal mitogenome (Hamari et al., 2002).

The total intergenic space found in the 12 genomes being compared range from 16,610 to 36,404 bp. Isolate GHA63, the largest *M. tenuissimus* mitogenome, has the smallest intergenic space (16,610 bp), while *Mar. scandens* has the largest intergenic space (36,404 bp) (Table 1). TRs are involved in expansion of intergenic space and have been associated with gene order changes (Aguileta et al., 2014). The number and types of TRs detected also vary with these species (Supplementary Excel File 1). *Mon. perniciosa* is the extreme with 60 TRs detected. *Mon. roreri* has 24 TRs while the closely related *M. palmivorus* has only 11 TRs, the lowest for any mitogenome studied here. Costa et al. (2012) also reported high repeat sequence in *Mon. perniciosa* compared to *Mon. roreri. M. tenuissimus* genomes that carry a single intron in *cox1* have 16 to 17 TRs while the 2 genomes carrying 3 *cox1* intron have 18 and 21 TRs. *M. crinis-equi* carries 24 TRs, including 4 within *cox1* introns (Supplementary Excel File 1). Although the types of TRs vary between *M. tenuissimus* isolates GHA63 and 79 and *M. crinis-equi*, these isolates share a pattern of increased TR numbers starting after *rps3* continuing to the following *trnR*. This pattern is less developed in the remaining *M. tenuissimus* isolates. Comparing the TRs between *rps3* and *trnR* region of the seven *M. tenuissimus* isolates shows that the overall number of TR regions for some of the isolates are increasing and the number of repeats for one TR region is also increasing (Supplementary Excel File 2). But at the same time, GHA79 has gained unique TR regions and lost a common TR region (Supplementary Excel File 2). Comparative analysis of the mitogenomes from three isolates of *L. edodes*, another *Agaricales* also revealed variable number TR regions (Kim et al., 2019). The authors proposed that the elongation of the repeat regions occurs through reciprocal incorporation of basic repeat units. Variations in TR number are thought to be generated by slipped-strand mispairing in the mitogenome (Mundy and Helbig, 2004) and/or genetic recombination (Nishizawa et al., 2000; Wang et al., 2015). In animals and plants, the TRs are responsible for mitogenome stability by insertion and deletion of the repeat array (Lobachev et al., 1998; Albert and Sellem, 2002). In fungi, repeat rearrangement occurs mostly through genetic recombination and contributes to mitogenome evolution (Aguileta et al., 2014). Whether selection acts upon the number and size of the repeat region in the mitogenome or results from some stochastic events related to erroneous DNA replication and repair needs further investigation.

As might be expected (Zardoya, 2020), the presence of uORF outside the introns also contributes to the differences in mitogenome sizes among the species studied. The large genome of *Mon. perniciosa* carries 40 accessory genes, many of which are part of an inserted linear plasmid (Formighieri et al., 2008), *Mon. roreri* carries 24 uORFs, *M. scandens* 7 uORFs, and *M. crinis-equi* 2 uORFs, *M. palmivorus* 14 uORFs, and *M. tenuissimus* 3 to 4 uORFs depending on the isolate. Most of the uORFs show extreme sequence variation within and between species (Supplementary Figure 7 and Supplementary Excel File 3). Most exceptions where similarities are significant occur between *Mon. perniciosa*, *Mon. roreri*, and *M. palmivorus*.

### GC Content

The average GC content of the 12 mitogenomes range between 26.58 and 32.04% (Table 1) which is in the higher range of values for fungal mitogenomes (average = 24.4 ± 7.3%, estimated from all fungal mitogenomes deposited in the NCBI organelle genome database) (Franco et al., 2017). Moreover, distribution of the GC content along the mitogenomes varied greatly among the species studied here in. The three closely related species, *Mon. perniciosa*, *Mon. roreri*, and *M. palmivorus*, have distinct patterns of GC content but do share some similarities (Supplementary Figure 8). All three species show above average GC content in association with the *rps3* and *rrnL*. *M. palmivorus* and *Mon. perniciosa* have significant areas of below average GC content associated with the *cox1* gene, its introns, and the block of uORFs following the *cox* 1 gene. This block of uORFs is missing in *Mon. roreri*. *Mon. perniciosa* and *Mon. roreri* also have distinct areas of low GC content associated with different blocks of uORFs not found in *M. palmivorus*. Otherwise, the shifts in GC content are muted in *M. palmivorus* compared to *Mon. roreri* and *Mon. perniciosa*. As might be expected, *M. tenuissimus* and *M. crinis-equi* show similar patterns of GC content. The pattern of GC content in *Mar. scandens*, with is higher overall GC content (32.04%), generally lacks the extremes observed in the *Mon. perniciosa*, *Mon. roreri*, and *M. palmivorus* grouping. Overall, patterns of low GC content are associated with the occurrence of introns containing ORFs and groupings of uORFs. There is also a tendency for areas of tRNA blocks to show above average GC content.

### The Ribosomal Protein Rps3

**FIGURE 5:**
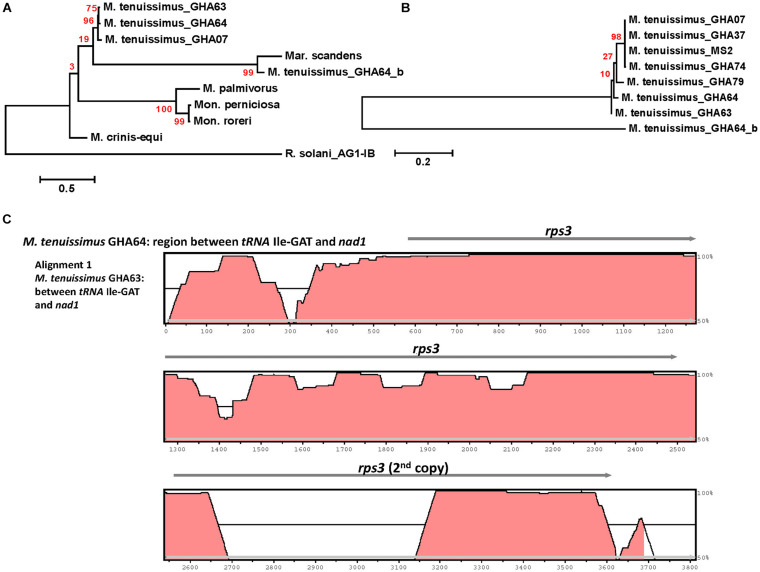
Molecular phylogenetic analysis of four cacao thread blight associated pathogens from Ghana (*M. tenuissimus*, *M. palmivorus*, *M. crinis-equi*, *Mar. scandens*) and the Western hemisphere frosty pod rot (*Mon. roreri*) and witches’ broom (*Mon. perniciosa*) pathogens of cacao based on the *rps3* gene. The analysis was based on amino-acid sequences of *rps3* homologs from each isolate with 81 distinct alignment positions and 1,000 rapid bootstrap inferences. **(A)** Three *M. tenuissimus* isolates with other five species. **(B)** Seven isolates of *M. tenuissimus*. Sequences were combined and aligned using ClustalW2 tool under default setting and the phylogenetic tree was reconstructed using the Maximum Likelihood method. The tree is drawn to scale, with branch lengths measured in the number of substitutions per site. Analyses were conducted in MEGA6 (Tamura et al., 2013). **(C)** Visualization of *rps3* gene region sequence alignment between *M. tenuissimus* isolate GHA64 and GHA63. The mVISTA program (http://genome.lbl.gov/vista/mvista/submit.shtml) was used to compare the section of mitochondrial genomes with default parameters in Shuffle-LAGAN mode and a sequence conservation profile was visualized in an mVISTA plot (Frazer et al., 2004).

Rps3 is the only ribosomal protein encoded in the fungal mitogenome and is involved in the assembly of the 37S ribosomal subunit (Seif et al., 2005). The *rps3* gene is extremely diverse in location and organization within mitogenomes and has a complex evolutionary history of acquisition by group I introns, loss of the intron, and establishment of *rps3* as a free-standing gene (Sethuraman et al., 2009). Korovesi et al. (2018) proposed two evolutionary routes for *rps3* gene accusation into the mitogenome, as a free-standing gene in the case of *Basidiomycota* and most of the yeasts or an anchored gene within the *omega* intron in case of *Pezizomycotina*. Complying with the model, at least one *rps3* is found in each mitogenome, existing as a free-standing gene (Figures 1, 3). The *rps3* genes in *Mon. perniciosa*, *Mon. roreri*, and *M. palmivorus* show sequence identity (>73%) (Figure 5) and are similarly located in front of the *rnL* gene (Figure 1). The *M. tenuissimus* and *M. crinis-equi rps3* genes also show sequence identity (>64%) (Figure 5A) and are similarly located prior to *nad1* (Figure 3). *M. tenuissimus* isolate GHA64 carries 2 copies of the *rps3* gene (68.9% identity), one being unique, and sharing sequence homology with the *Mar. scandens rps3* (73.5% identity) but not the other isolates of *M. tenuissimus* (Figures 5A,B). Alignment of the *rps3* gene region between *M. tenuissimus* isolate GHA64 and GHA63 shows an insertion of a 479 bp segment in the GHA64 mitogenome, which is the part of the second *rps3* copy (Figure 5C). The phylogenetic relationships using the 14 core proteins are largely mimicked by relationships between the *rps3* gene comparisons (Figures 4, 5). The *rps3* gene provides an alternative to the whole mitogenome as a molecular marker for phylogenic study. The *rps3* gene was used for phylogenetic analysis of *Colletotrichum* species (Pszczółkowska et al., 2020).

### Introns and Accessory Mitochondrial Genes

Intron dynamics play an important role in altering organization and size of fungal mitogenomes (Himmelstrand et al., 2014; Li et al., 2021). Group I and group II introns are commonly found, among other places, in organelles of higher eukaryotes (Haugen et al., 2005). Often considered selfish DNA, they can act as ribozymes catalyzing their own splicing from a precursor RNA and restoring the translational reading frames of their host genes. Group I introns, which are mobile due to the activity of HEGs, are abundant in fungal mitogenomes (Lang et al., 2007). In some instances, HEGs function as mobile elements, moving independently from their host intron to new locations (Edgell, 2009). These endonucleases, having settled within introns, provide a valuable source of genetic information. The mitogenomes of the six *Marasmiaceae* family members harbor varying numbers of introns, all corresponding to group I. Most of the introns found in these genomes are group 1A and 1B introns. *Mon. perniciosa* also carries group 1C introns and *M. crinis-equi* carries group 1D introns, while *Mar. scandens* and *Mon. roreri* carry both group 1C and 1D introns (Supplementary Excel File 1). Introns in mitogenomes can have direct biological consequences. Possibly, the best example of this is the involvement of group I introns blocking the main mutation involved in the resistance against Quinone outside Inhibitor (QoIs) fungicides (Grasso et al., 2006). Recently, Cinget and Bélanger (2020) identified 216 novel group 1D introns involved in QoIs resistance and hypothesized that mobility of the intron across fungal mitogenomes influences the ability to develop resistance to QoIs. *Mon. roreri* has 14 ORFs within 13 introns, *Mon. perniciosa* 10 ORFs within 13 introns, *Mar. scandens* 8 ORFs within 9 introns, and *M. crinis-equi* and *M. palmivorus* 6 ORFs within 7 introns. *M. tenuissimus* isolates GHA63 and GHA79 carry 3 ORFs within 3 introns all within the *Cox1* gene while the remaining isolates carry only one ORF within one intron within the *cox1* gene (Figure 3). The intronic ORFs identified regardless of species encode GIY-YIG or LAGLIDADG domain-containing HEGs (Supplementary Excel File 1).

**FIGURE 6:**
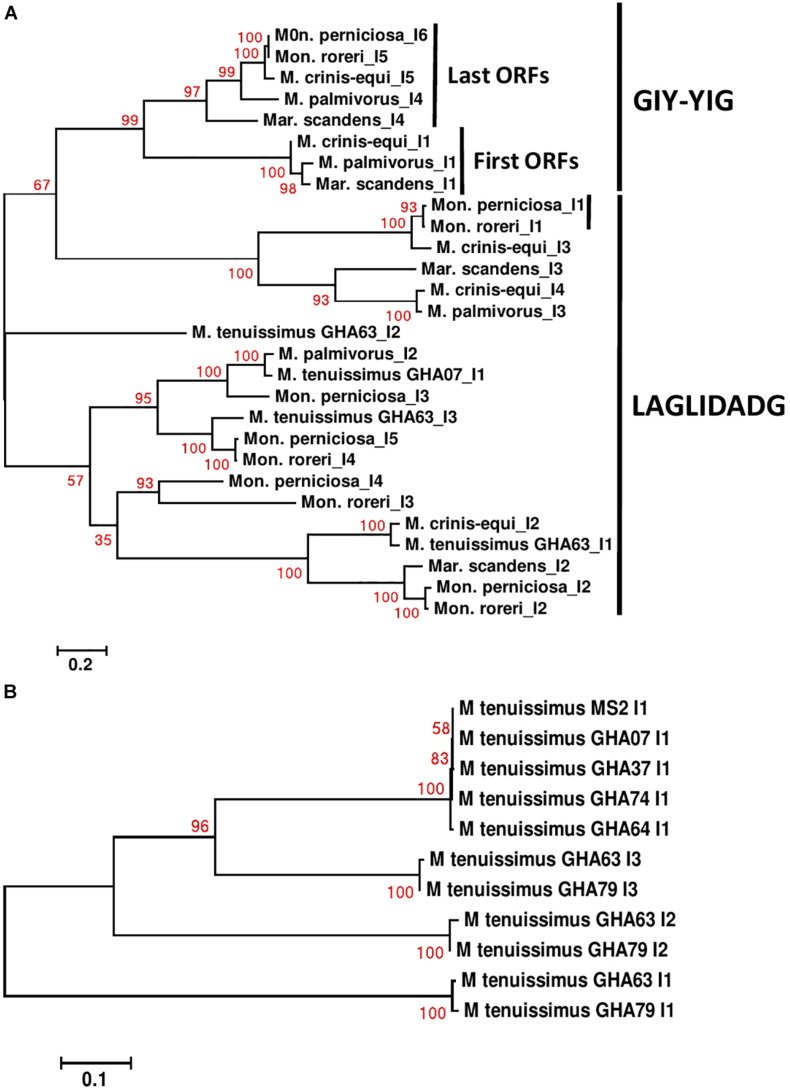
Molecular phylogenetic analysis of *cox1* introns of four cacao thread blight associated pathogens from Ghana (*M. tenuissimus*, *M. palmivorus*, *M. crinis-equi*, *Mar. scandens*) and the Western hemisphere frosty pod rot (*Mon. roreri*) and witches’ broom (*Mon. perniciosa*) pathogens of cacao. The analysis was based on nucleotide sequences of *cox 1* introns from each isolate with 1,566 distinct alignment positions and 1,000 rapid bootstrap inferences. **(A)** Two *M. tenuissimus* isolates with other five species. **(B)** Seven isolates of *M. tenuissimus*. Sequences were combined and aligned using ClustalW2 tool under default setting and the phylogenetic tree was reconstructed using the Maximum Likelihood method. The tree is drawn to scale, with branch lengths measured in the number of substitutions per site. Analyses were conducted in MEGA6 (Tamura et al., 2013).

Specific sequence comparisons can be made among the intronic ORFs found in the *Marasmiaceae* mitogenomes due to their limited numbers (Supplementary Figure 9). As expected, the intronic ORFs show substantial sequence differences depending on whether they are GIY-YIG or LAGLIDADG domain-containing HEGs and clusters separately (Figure 6A). For *M. tenuissimus*, the five isolates carrying a single *cox1* intronic ORF show close similarity (>99% identity) while the 3 *cox1* intronic ORFs of isolates 63 and 79 show close similarity (>99% identity) in a positionally dependent manner (Figure 6B). As seen in other comparisons, the genetic relationships among these intronic ORFs across species, in some cases, also show specificity as to position within specific core genes (Huang et al., 2021). For example, the terminal GIY-YIG containing intronic ORF in *cox1* shows sequence similarity (>95%) across all species except *M. tenuissimus* (Figure 6A). The positional relationships among intronic ORFs are not consistent, as is expected considering the variable number of intronic ORFs found depending on the species. The terminal intronic ORF (LAGLIDADG type) of the *cox1* gene in *M. tenuissimus* (*orf322*-I3) is similar to (>92% sequence similarity) the next to last ORFs (*orf332*-I4 and I5) in the more distantly related *Mon. roreri*, and *Mon. perniciosa*. Another example occurs between the middle LAGLIDADG type intronic ORF of *cox1* in *M. crinis-equi* (*orf388*-I3) and the initial *cox1* intronic ORF in *Mon. roreri* (*orf415*-I1), and *Mon. perniciosa* (*orf422*-I1). The relationships between LAGLIDADG type intronic ORFs appear more focused, involving smaller subsets within the six species. These relationships do not always involve the *Moniliophthora*, for example, the next to last intronic ORF (LAGLIDADG type) of the *cox1* gene in *M. crinis-equi* (*orf278*-I4) and *M. palmivorus* (*orf278*-I3) have 99% sequence similarity not shared with any *Moniliophthora* intronic ORF. The single GIY-YIG containing intronic ORF found in *cob* also shows sequence similarity in *M. palmivorus*, *Mar. scandens*, *M. crinis-equi*, and *Mon. roreri* (Supplementary Figure 9). The *cob* genes in *M. tenuissimus* and *Mon. perniciosa* do not carry a GIY-YIG containing intron. Despite the close sequence similarities between species for many of the intronic ORFs, all species compared except *M. crinis-equi* have at least one intronic ORF that has minimal sequence similarity (% < 40) with any other intronic ORF. The middle cox1 intronic ORF of *M. tenuissimus* isolate GHA63 (*orf311*-I2) has minimal sequence homology with any cox1 intronic ORF identified and instead shows its closest sequence homology with a *Mon. roreri* cob intronic ORF (*orf357*). The sequence and positional similarities and dissimilarities found for many of the *Marasmiaceae* intronic ORFs indicates a shared ancestral origin for many and varied histories of expansion and contraction dependent on the species, which is typical for mitogenomes (Megarioti and Kouvelis, 2020; Mukhopadhyay and Hausner, 2021). The intronic ORFs also share significant homologies with mitogenome intronic ORFs of unrelated fungal species (Supplementary Excel File 2), often outside the basidiomycetes, something commonly cited as evidence of introgression between diverse species. Similar intron dynamics were observed within the *Agaricales* (Huang et al., 2021).

## Conclusion

The mitogenomes of the six *Marasmiaceae* species compared display a wide range of genome sizes and composition. The *Mon. perniciosa* and *Mon. roreri* mitogenomes are the most complex, having the largest sizes and numbers of both intronic ORFs and uORFs. The most commonly encountered TBD-associated pathogen in Ghana, *M. tenuissimus*, has the smallest mitogenomes. The well-defined gene order patterns and core gene sequence similarities group *M. tenuissimus* with *M. crinis-equi*, and *M. palmivorus* with *Mon. roreri* and *Mon. perniciosa*, leaving *Mar. scandens* as an outlier among the species studied. Most of the intronic ORFs of the TBD-associated pathogens share sequence homology with intronic ORFs found in the *Mon. perniciosa* and *Mon. roreri* mitogenomes, although exceptions exist. Together, the reduced numbers of intronic ORFs found in the TBD-associated pathogens compared to the *Moniliophthoras*, the common homologies of most intronic ORFs among the species, and the common occurrence of uORFs lacking shared homologies provide evidence of independent expansion/contraction events and sequence diversification in each species depending on their physiological and developmental needs. The freestanding *rps3* gene is a promising marker that may also be useful in inferring phylogenies within *Marasmiaceae*. Among the seven mitogenomes of *M. tenuissimus* sequenced, there is variation in both size and sequences pointing to three distinct phylactic groups within the species. This is the first mitogenome phylogeny of the genus *Marasmius*, and results highlight the potential for future systematics changes in *Marasmius* and crypticism in *M. tenuissimus.*

## Data Availability Statement

The mitogenome sequence data presented in the study are deposited at NCBI GenBank under the accession numbers MZ615343–MZ615352.

## Author Contributions

BB, SA, and LM provided intellectual and editorial comments. SA and BB conceived and designed the experiments and wrote the first draft manuscript. IA-A and EK-A isolation of fungal isolates. SA performed the experiments. SA and JS analyzed the data. All authors contributed to the manuscript revision, read, and approved the submitted version.

## Conflict of Interest

The authors declare that the research was conducted in the absence of any commercial or financial relationships that could be construed as a potential conflict of interest.

## Publisher’s Note

All claims expressed in this article are solely those of the authors and do not necessarily represent those of their affiliated organizations, or those of the publisher, the editors and the reviewers. Any product that may be evaluated in this article, or claim that may be made by its manufacturer, is not guaranteed or endorsed by the publisher.

## Footnotes

1
https://github.com/aquaskyline/SOAPdenovo2

2
https://megasun.bch.umontreal.ca/RNAweasel/

3
https://www.ncbi.nlm.nih.gov/genome/organelle/

4
http://genome.lbl.gov/vista/mvista/submit.shtml

## References

[B1] Aguileta G. De Vienne D. M. Ross O. N. Hood M. E. Giraud T. Petit E. (2014). High variability of mitochondrial gene order among fungi. *Genome Biol. Evol.* 6 451–465. 10.1093/gbe/evu028 24504088 PMC3942027

[B2] Albert B. Sellem C. H. (2002). Dynamics of the Mitochondrial Genome during *Podospora Anserina* Aging. *Curr. Genet.* 40 365–373. 10.1007/s00294-002-0275-1 11919675

[B3] Ali S. S. Asman A. Shao J. Balidion J. F. Strem M. D. Puig A. S. (2020). Genome and transcriptome analysis of the latent pathogen *Lasiodiplodia theobromae*, an emerging threat to the cacao industry. *Genome* 63 37–52. 10.1139/gen-2019-0112 31580730

[B4] Ali S. S. Shao J. Lary D. J. Kronmiller B. A. Shen D. Strem M. D. (2017). *Phytophthora megakarya* and *Phytophthora palmivora*, closely related causal agents of cacao black pod rot, underwent increases in genome sizes and gene numbers by different mechanisms. *Genome Biol. Evol.* 9 536–557. 10.1093/gbe/evx021 28186564 PMC5381587

[B5] Ali S. S. Shao J. Strem M. D. Phillips-Mora W. Zhang D. Meinhardt L. W. (2015). Combination of RNAseq and SNP nanofluidic array reveals the center of genetic diversity of cacao pathogen *Moniliophthora roreri* in the upper Magdalena Valley of Colombia and its clonality. *Frontiers Microbiol.* 6:850. 10.3389/fmicb.2015.00850 26379633 PMC4550789

[B6] Altschul S. F. Gish W. Miller W. Myers E. W. Lipman D. J. (1990). Basic local alignment search tool. *J. Mol. Biol.* 215 403–410. 10.1016/S0022-2836(05)80360-2 2231712

[B7] Amoako-Attah I. Akrofi A. Y. Hakeem R. Bin, Asamoah M. Kumi-Asare E. (2016). White thread blight disease caused by *Marasmiellus scandens* (Massee) Dennis Reid on cocoa and its control in Ghana. *Afr. J. Agric. Res.* 11 5064–5070. 10.5897/AJAR2016.11681

[B8] Amoako-Attah I. Ali S. S. Aime M. C. Odamtten G. T. Cornelius E. Nyaku S. T. (2020). Identification and Characterization of fungi causing thread blight diseases on cacao in Ghana. *Plant Dis.* 104 3033–3042. 10.1094/PDIS-03-20-0565-RE 32822261

[B9] Benson G. (1999). Tandem repeats finder: a program to analyze DNA sequences. *Nucleic Acids Res.* 27 573–580. 10.1093/nar/27.2.573 9862982 PMC148217

[B10] Bullerwell C. E. Lang B. F. (2005). Fungal evolution: the case of the vanishing mitochondrion. *Curr. Opin. Microbiol.* 8 362–369. 10.1016/j.mib.2005.06.009 15993645

[B11] Burt A. Koufopanou V. (2004). Homing endonuclease genes: the rise and fall and rise again of a selfish element. *Curr. Opin. Genet. Dev.* 14 609–615. 10.1016/j.gde.2004.09.010 15531154

[B12] Cinget B. Bélanger R. R. (2020). Discovery of new group ID introns leads to creation of subtypes and link to an adaptive response of the mitochondrial genome in fungi. *RNA Biol.* 17 1252–1260. 10.1080/15476286.2020.1763024 32449459 PMC7595605

[B13] Conesa A. Götz S. García-Gómez J. M. Terol J. Talón M. Robles M. (2005). Blast2GO: a universal tool for annotation, visualization and analysis in functional genomics research. *Bioinformatics* 21 3674–3676. 10.1093/bioinformatics/bti610 16081474

[B14] Costa G. G. L. Cabrera O. G. Tiburcio R. A. Medrano F. J. Carazzolle M. F. Thomazella D. P. T. (2012). The mitochondrial genome of *Moniliophthora roreri*, the frosty pod rot pathogen of cacao. *Fungal Biol.* 116 551–562. 10.1016/j.funbio.2012.01.008 22559916

[B15] Darzentas N. (2010). Circoletto: visualizing sequence similarity with Circos. *Bioinformatics* 26 2620–2621. 10.1093/bioinformatics/btq484 20736339

[B16] Desjardin D. E. Perry B. A. (2017). The gymnopoid fungi (*Basidiomycota, Agaricales*) from the Republic of São Tomé and Príncipe, West Africa. *Mycosphere* 8 1317–1391. 10.5943/mycosphere/8/9/5

[B17] Díaz-Valderrama J. R. Aime M. C. (2016). The cacao pathogen *Moniliophthora roreri* (*Marasmiaceae*) possesses biallelic A and B mating loci but reproduces clonally. *Heredity* 116 491–501. 10.1038/hdy.2016.5 26932308 PMC4868271

[B18] Edgell D. R. (2009). Selfish DNA: homing endonucleases find a home. *Curr. Biol.* 19 R115–R117. 10.1016/j.cub.2008.12.019 19211047

[B19] Evans H. C. Holmes K. A. Reid A. P. (2003). Phylogeny of the frosty pod rot pathogen of cocoa. *Plant Pathol.* 52 476–485. 10.1046/j.1365-3059.2003.00867.x

[B20] Formighieri E. F. Tiburcio R. A. Armas E. D. Medrano F. J. Shimo H. Carels N. (2008). The mitochondrial genome of the phytopathogenic basidiomycete *Moniliophthora perniciosa* is 109 kb in size and contains a stable integrated plasmid. *Mycol. Res.* 112 1136–1152. 10.1016/j.mycres.2008.04.014 18786820

[B21] Franco M. E. E. López S. M. Y. Medina R. Lucentini C. G. Troncozo M. I. Pastorino G. N. (2017). The mitochondrial genome of the plant-pathogenic fungus *Stemphylium lycopersici* uncovers a dynamic structure due to repetitive and mobile elements. *PLoS One* 12:e0185545. 10.1371/journal.pone.0185545 28972995 PMC5626475

[B22] Frazer K. A. Pachter L. Poliakov A. Rubin E. M. Dubchak I. (2004). VISTA: computational tools for comparative genomics. *Nucleic Acids Res.* 32 W273–W279. 10.1093/nar/gkh458 15215394 PMC441596

[B23] Gaire S. P. Zhou X.-G. Jo Y.-K. (2020). Sterile White Basidiomycete Fungus *Marasmius graminum*: A New Pathogen Causing Seedling Blight in Rice. *Plant Dis.* 105:702. 10.1094/PDIS-05-20-1136-PDN 33021914

[B24] Grasso V. Sierotzki H. Garibaldi A. Gisi U. (2006). Characterization of the cytochrome b gene fragment of *Puccinia* species responsible for the binding site of QoI fungicides. *Pestic. Biochem. Physiol.* 84 72–82. 10.1016/j.pestbp.2005.05.005

[B25] Hamari Z. Juhász Á Kevei F. (2002). Role of mobile introns in mitochondrial genome diversity of fungi. *Acta Microbiol. Immunol. Hung.* 49 331–335. 10.1556/amicr.49.2002.2-3.22 12109166

[B26] Haugen P. Simon D. M. Bhattacharya D. (2005). The natural history of group I introns. *Trends Genet.* 21 111–119. 10.1016/j.tig.2004.12.007 15661357

[B27] Hausner G. (2003). “Fungal Mitochondrial Genomes, Plasmids and Introns,” in *Fungal Genomics*, Vol. 3 eds Arora D. K. Khachatourians G. G. (New York, NY: Elsevier Science), 101–131. 10.1016/S1874-5334(03)80009-6

[B28] Himmelstrand K. Olson A. Durling M. B. Karlsson M. Stenlid J. (2014). Intronic and Plasmid-Derived Regions Contribute to the Large Mitochondrial Genome Sizes of Agaricomycetes. *Curr. Genet.* 60 303–313. 10.1007/s00294-014-0436-z 25011705 PMC4201751

[B29] Huang W. Feng H. Tu W. Xiong C. Jin X. Li P. (2021). Comparative mitogenomic analysis reveals dynamics of intron within and between *Tricholoma* species and phylogeny of Basidiomycota. *Front. Genet.* 12:534871. 10.3389/fgene.2021.534871 33659021 PMC7917209

[B30] Kaniak-Golik A. Skoneczna A. (2015). Mitochondria–Nucleus Network for Genome Stability. *Free Radic. Biol. Med.* 82 73–104. 10.1016/j.freeradbiomed.2015.01.013 25640729

[B31] Kim S. Song Y. Ha B. Moon Y. J. Kim M. Ryu H. (2019). Variable Number Tandem Repeats in the Mitochondrial DNA of *Lentinula edodes*. *Genes* 10:542. 10.3390/genes10070542 31319586 PMC6679062

[B32] Kirschner R. Lee I.-S. Chen C.-J. (2013). Ovularia puerariae Sawada is the hyphomycetous anamorph of a new *Marasmius* species on living leaves of kudzu (*Pueraria montana*, Fabaceae). *Mycologia* 105 781–792. 10.3852/12-285 23360973

[B33] Kobayashi M. Matsuo Y. Takimoto A. Suzuki S. Maruo F. Shoun H. (1996). Denitrification, a novel type of respiratory metabolism in fungal mitochondrion. *J. Biol. Chem.* 271 16263–16267. 10.1074/jbc.271.27.16263 8663075

[B34] Koch R. A. Lodge D. J. Sourell S. Nakasone K. McCoy A. G. Aime M. C. (2018). Tying up Loose Threads: Revised Taxonomy and Phylogeny of an Avian-Dispersed Neotropical Rhizomorph-Forming Fungus. *Mycol. Prog.* 17 989–998. 10.1007/s11557-018-1411-8

[B35] Korovesi A. G. Ntertilis M. Kouvelis V. N. (2018). Mt-rps3 is an ancient gene which provides insight into the evolution of fungal mitochondrial genomes. *Mol. Phylogenet. Evol.* 127 74–86. 10.1016/j.ympev.2018.04.037 29763662

[B36] Kües U. Navarro-González M. (2010). Mating-type orthologous genes in the primarily homothallic *Moniliophthora perniciosa*, the causal agent of witches’ broom disease in cacao. *J. Basic Microbiol.* 50 442–451. 10.1002/jobm.201000013 20586074

[B37] Kulik T. Van Diepeningen A. D. Hausner G. (2020). The Significance of Mitogenomics in Mycology. *Front. Microbiol*. 11:628579. 10.3389/fmicb.2020.628579 33488569 PMC7817700

[B38] Lang B. F. Laforest M.-J. Burger G. (2007). Mitochondrial introns: a critical view. *Trends in Genet.* 23 119–125. 10.1016/j.tig.2007.01.006 17280737

[B39] Larkin M. A. Blackshields G. Brown N. P. Chenna R. McGettigan P. A. McWilliam H. (2007). Clustal W and Clustal X version 2.0. *Bioinformatics* 23 2947–2948. 10.1093/bioinformatics/btm404 17846036

[B40] Li Q. Li L. Feng H. Tu W. Bao Z. Xiong C. (2021). Characterization of the Complete Mitochondrial Genome of Basidiomycete Yeast *Hannaella Oryzae*: Intron Evolution, Gene Rearrangement, and Its Phylogeny. *Front. Microbiol.* 12:646567. 10.3389/fmicb.2021.646567 34122362 PMC8193148

[B41] Lobachev K. S. Shor B. M. Tran H. T. Taylor W. Keen J. D. Resnick M. A. (1998). Factors Affecting Inverted Repeat Stimulation of Recombination and Deletion in *Saccharomyces Cerevisiae*. *Genet* 148 1507–1524. 10.1093/genetics/148.4.1507 9560370 PMC1460095

[B42] Lohse M. Drechsel O. Bock R. (2007). OrganellarGenomeDRAW (OGDRAW): a tool for the easy generation of high-quality custom graphical maps of plastid and mitochondrial genomes. *Curr. Genet.* 52 267–274. 10.1007/s00294-007-0161-y 17957369

[B43] Massee G. (1910). *Fungi exotici: XI. Bulletin of Miscellaneous Information (Royal Botanic Gardens, Kew).* Berlin: Springer, 249–253. 10.2307/4111851

[B44] Megarioti A. H. Kouvelis V. N. (2020). The coevolution of fungal mitochondrial introns and their Homing Endonucleases (GIY-YIG and LAGLIDADG). *Genome Biol. Evol.* 12 1337–1354. 10.1093/gbe/evaa126 32585032 PMC7487136

[B45] Mukhopadhyay J. Hausner G. (2021). Organellar Introns in Fungi, Algae, and Plants. *Cells* 10:2001. 10.3390/cells10082001 34440770 PMC8393795

[B46] Mundy N. I. Helbig A. J. (2004). Origin and Evolution of Tandem Repeats in the Mitochondrial DNA Control Region of Shrikes (*Lanius Spp*.). *J. Mol. Evol.* 59 250–257. 10.1007/s00239-004-2619-6 15486698

[B47] Nishizawa S. Kubo T. Mikami T. (2000). Variable Number of Tandem Repeat Loci in the Mitochondrial Genomes of Beets. *Curr. Genet.* 37 34–38. 10.1007/s002940050005 10672442

[B48] Opoku I. Y. Assuah M. K. Domfeh O. (2007). Manual for the identification and control of diseases of cocoa. *Cocoa Res. Instit. Ghana Ghana Cocoa Board Ghana Technical Bull* 16 18–19.

[B49] Pszczółkowska A. Androsiuk P. Jastrzêbski J. P. Paukszto L. Okorski A. (2020). Rps3 as a Candidate Mitochondrial Gene for the Molecular Identification of Species from the *Colletotrichum Acutatum* Species Complex. *Genes* 11:552. 10.3390/genes11050552 32422999 PMC7290925

[B50] Repar J. Warnecke T. (2017). Mobile introns shape the genetic diversity of their host genes. *Genetics* 205 1641–1648. 10.1534/genetics.116.199059 28193728 PMC5378118

[B51] Richter C. (1992). Reactive oxygen and DNA damage in mitochondria. *Mut. Res. DNAging* 275 249–255. 10.1016/0921-8734(92)90029-O 1383767

[B52] Sandor S. Zhang Y. Xu J. (2018). Fungal Mitochondrial Genomes and Genetic Polymorphisms. *Appl. Microbiol. Biotechnol.* 102 9433–9448. 10.1007/s00253-018-9350-5 30209549

[B53] Seif E. Leigh J. Liu Y. Roewer I. Forget L. Lang B. F. (2005). Comparative Mitochondrial Genomics in Zygomycetes: Bacteria-like RNase P RNAs, Mobile Elements and a Close Source of the Group I Intron Invasion in Angiosperms. *Nucleic Acids Res.* 33 734–744. 10.1093/nar/gki199 15689432 PMC548346

[B54] Sethuraman J. Majer A. Iranpour M. Hausner G. (2009). Molecular evolution of the mtDNA encoded rps3 gene among filamentous ascomycetes fungi with an emphasis on the ophiostomatoid fungi. *J. Mol. Evol.* 69 372–385. 10.1007/s00239-009-9291-9 19826748

[B55] Stoddard B. L. (2011). Homing endonucleases: from microbial genetic invaders to reagents for targeted DNA modification. *Structure* 19 7–15. 10.1016/j.str.2010.12.003 21220111 PMC3038549

[B56] Su H. J. Thseng F. M. Chen J. S. Ko W.-H. (2011). Production of volatile substances by rhizomorphs of *Marasmius crinisequi* and its significance in nature. *Fungal Divers.* 49 199–202. 10.1007/s13225-010-0084-7

[B57] Tamura K. Stecher G. Peterson D. Filipski A. Kumar S. (2013). MEGA6: molecular evolutionary genetics analysis version 6.0. *Mol. Biol. Evol.* 30 2725–2729. 10.1093/molbev/mst197 24132122 PMC3840312

[B58] Torriani S. F. F. Penselin D. Knogge W. Felder M. Taudien S. Platzer M. (2014). Comparative Analysis of Mitochondrial Genomes from Closely Related Rhynchosporium Species Reveals Extensive Intron Invasion. *Fungal Genet. Biol*. 62 34–42. 10.1016/j.fgb.2013.11.001 24240058

[B59] Valach M. Burger G. Gray M. W. Lang B. F. (2014). Widespread Occurrence of Organelle Genome-Encoded 5S rRNAs Including Permuted Molecules. *Nucleic Acids Res.* 42 13764–13777. 10.1093/nar/gku1266 25429974 PMC4267664

[B60] Wang X. Liu N. Zhang H. Yang X.-J. Huang Y. Lei F. (2015). Extreme Variation in Patterns of Tandem Repeats in Mitochondrial Control Region of Yellow-Browed Tits (*Sylviparus Modestus*, Paridae). *Sci. Rep.* 5 1–9. 10.1038/srep13227 26288099 PMC4541255

[B61] Wilson A. J. Xu J. (2012). Mitochondrial inheritance: diverse patterns and mechanisms with an emphasis on fungi. *Mycol* 3 158–166.

[B62] Wu B. Hao W. (2014). Horizontal transfer and gene conversion as an important driving force in shaping the landscape of mitochondrial introns. *G3 Genes Genom. Genet.* 4 605–612. 10.1534/g3.113.009910 24515269 PMC4059233

[B63] Wu P. Yao T. Ren Y. Ye J. Qing Y. Li Q. (2021). Evolutionary Insights into Two Widespread Ectomycorrhizal Fungi (Pisolithus) from Comparative Analysis of Mitochondrial Genomes. *Front. Microbiol*. 12:583129. 10.3389/fmicb.2021.583129 34290675 PMC8287656

[B64] Xu J. Li H. (2015). Current perspectives on mitochondrial inheritance in fungi. *Cell Health Cytoskelet.* 7 143–154. 10.2147/CHC.S59508

[B65] Xu J. Wang P. (2015). Mitochondrial inheritance in basidiomycete fungi. *Fungal Biol. Rev.* 29 209–219. 10.1016/j.fbr.2015.02.001

[B66] Zardoya R. (2020). Recent advances in understanding mitochondrial genome diversity. *F1000Research* 9:21490.1. 10.12688/f1000research.21490.1 32399193 PMC7194472

[B67] Zhang Y. Yang G. Fang M. Deng C. Zhang K.-Q. Yu Z. (2020). Comparative analyses of mitochondrial genomes provide evolutionary insights into nematode-trapping fungi. *Front. Microbiol.* 11:617. 10.3389/fmicb.2020.00617 32351475 PMC7174627

