# Supplementary material for: Mitochondrial Genomics of Six Cacao Pathogens From the Basidiomycete Family Marasmiaceae
Source: Front Microbiol. 2021 Oct 28;12:752094. doi: 10.3389/fmicb.2021.752094 (PMC8581569; doi:10.3389/fmicb.2021.752094)
Supplement: Supplementary file 1 [file Data_Sheet_1.PDF]

Supplementary Figures

Mitochondrial genomics of six cacao pathogens from the basidiomycete family *Marasmiaceae*

Shahin S. Ali[1,2], Ishmael Amoako-Attah[3], Jonathan Shao[4], Eric A. Kumi[3], Lyndel W. Meinhardt[1] and Bryan A. Bailey[1*]

[1]Sustainable Perennial Crops Laboratory, USDA/ARS, Beltsville Agricultural Research Center-West, Beltsville, MD 20705, USA.
[2]Department of Viticulture and Enology, University of California, Davis, CA 95616.
[3]Cocoa Research Institute of Ghana, Akim New-Tafo, Ghana.
[4]USDA/ARS, Northeast Area, Beltsville, MD 20705, USA.
*Address correspondence to: Bryan A. Bailey, bryan.bailey@usda.gov

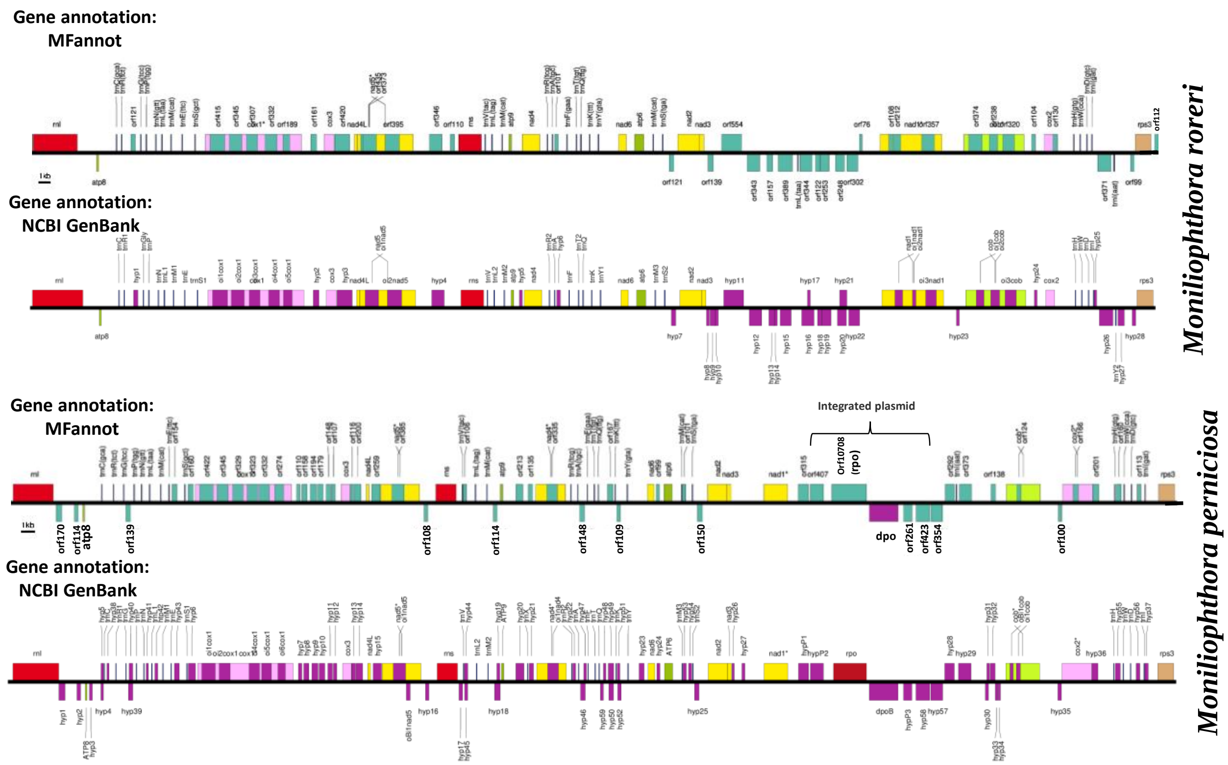

Supplementary Figure S1 Comparison of mitogenome structure annotation performed with MFannot and that obtained from NCBI for *Moniliophthora roreri* and *Moniliophthora perniciosa*. Mitochondrial gene annotation of *Mon. roreri* (Genbank no. HQ259115.1), *Mon. perniciosa* (Genbank no. AY376688.1) were performed with MFannot using the NCBI translation Table 4 and gene annotations were also obtained from NCBI as previously reported (Costa et al., 2012; Formighieri et al., 2008), then the physical maps were created with OrganellarGenome-DRAW (OGDRAW) v 1.2.

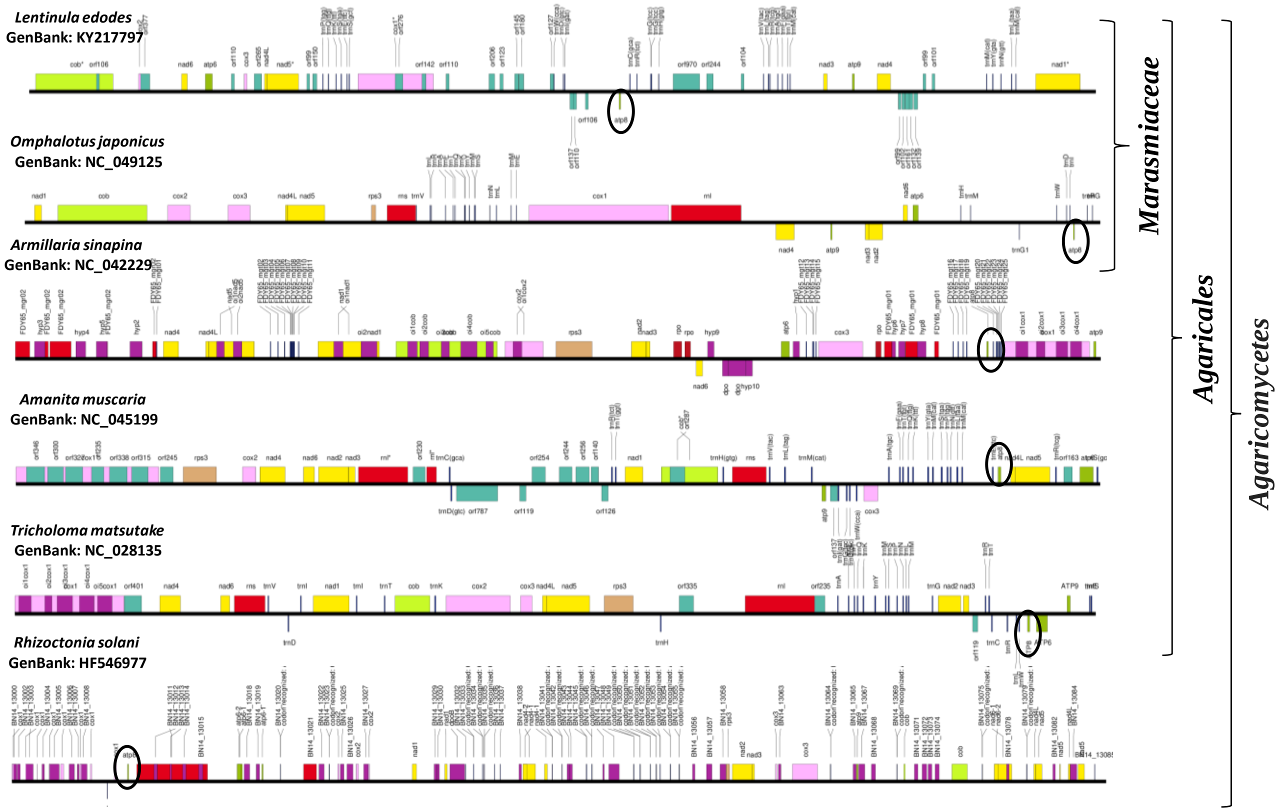

Supplementary Figure S2 Gene order and orientation of *atp8* gene in the mitogenome structure of fungus within the class *Agaricomycetes*. Whole mitochondrial genome sequence involved are *Lentinula edodes* (Genbank no. KY217797) *and Omphalotus japonicas* (Genbank no. NC_049125) within family *Marasmiaceae*; *Armillaria sinapina* (GenBank no. NC_042229), *Amanita muscaria* (GenBank no. NC_045199) and *Tricholoma matsutake* (GenBank no. NC_028135) within order *Agaricales*; and *Rhizoctonia solani* (GenBank no. HF546977) within class *Agaricomycetes*. Mitochondrial gene annotations were obtained from NCBI and the physical map of the mitogenomes were created with OrganellarGenome-DRAW (OGDRAW) v 1.2. The *atp8* gene were highlighted with black circle.

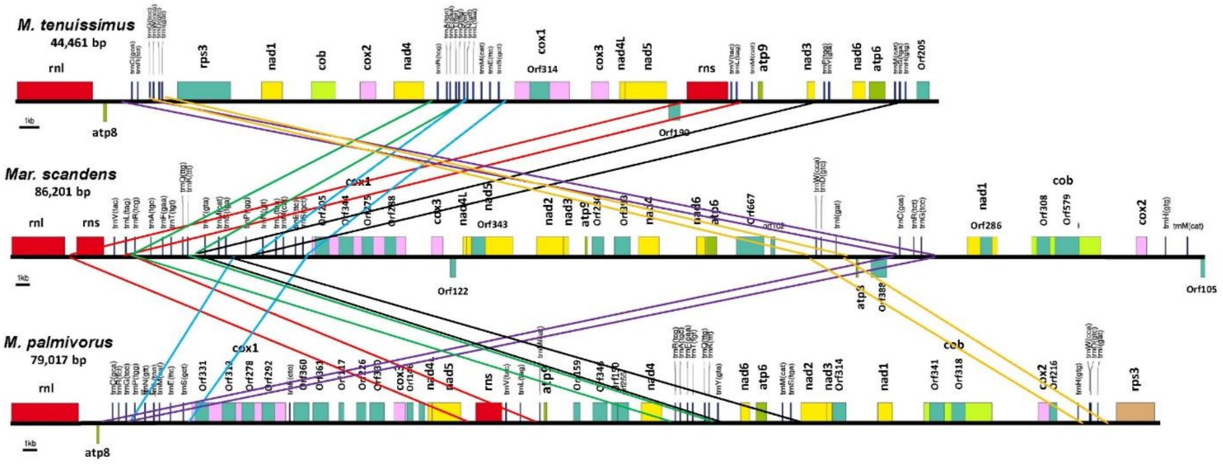

Supplementary Figure S3 Interspecies variation in tRNA block rearrangement in the mitogenome of three cacao thread blight associated pathogens from Ghana, all basidiomycetes within the *Marasmiaceae* family. Whole mitochondrial genome sequence involved are *Marasmius tenuissimus* (GHA07), *Marasmiellus scandens* (GHA19), and *Marasmius palmivorus* (GHA12). Mitochondrial gene annotation was performed with MFannot using the NCBI translation Table 4, and the physical map of the mitogenomes were created with OrganellarGenome-DRAW (OGDRAW) v 1.2. The tRNAs within same color lines represent one block.

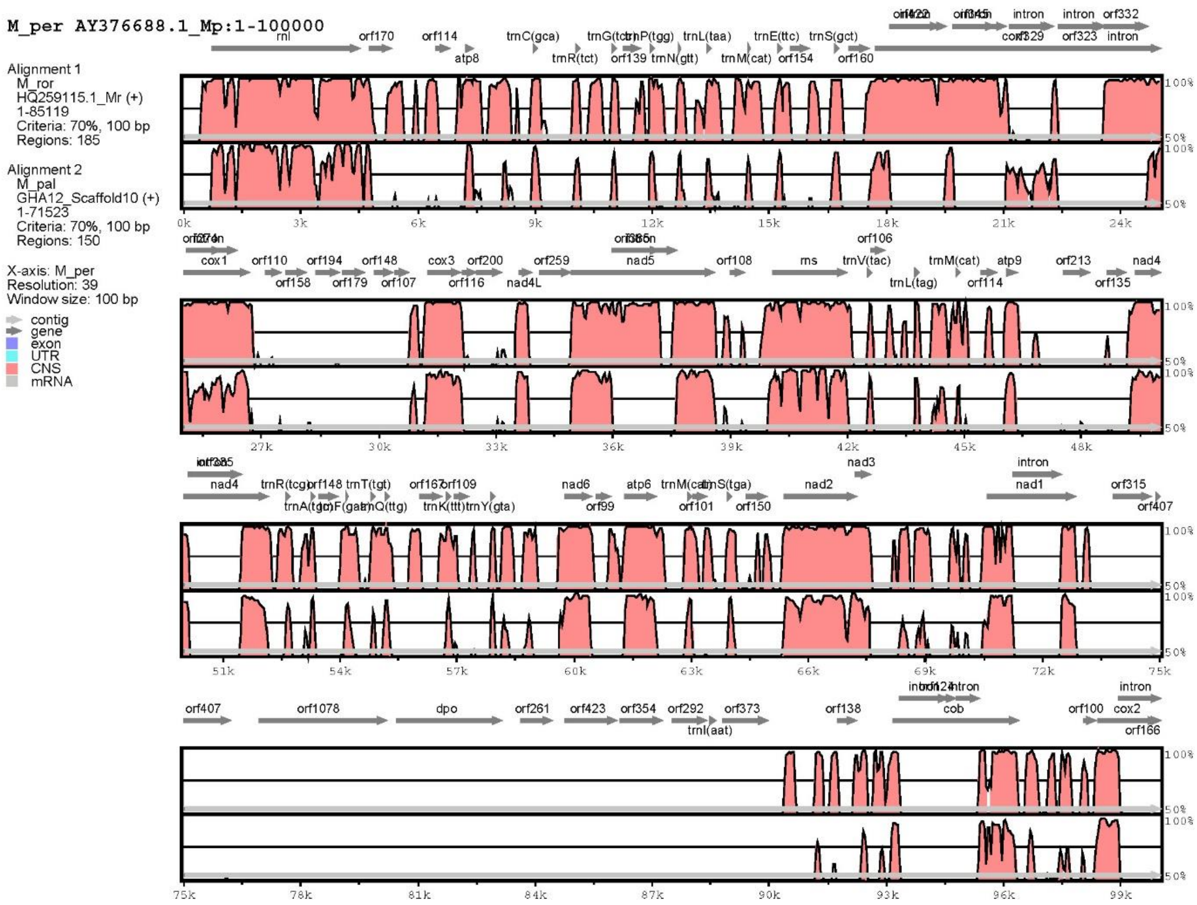

Supplementary Figure S4 Visualization of mitochondrial genomes sequence alignment between *Moniliophthora perniciosa* (Genbank no. AY376688.1), *Moniliophthora roreri* (Genbank no. HQ259115.1) and *Marasmius palmivorus* (GHA12). The mVISTA program (http://genome.lbl.gov/vista/mvista/submit.shtml) was used to compare the complete mitochondrial genomes of *Mon. perniciosa* with those of *Mon. roreri* and *Mar. palmivorus*, taking the annotation of the mitochondrial genome of *Mon. perniciosa* as a reference. Default parameters were utilized to align the genomes in Shuffle-LAGAN mode and a sequence conservation profile was visualized in an mVISTA plot (Frazer et al., 2004).

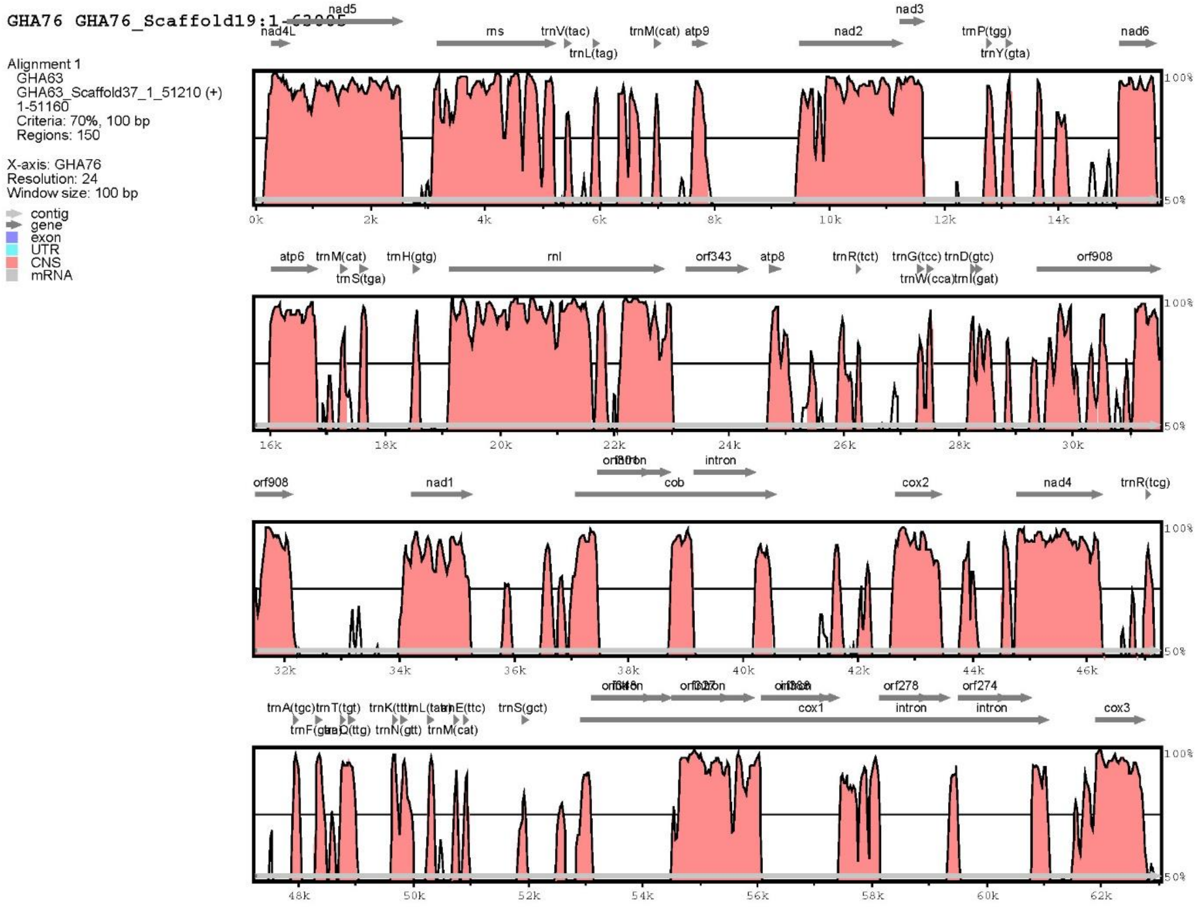

Supplementary Figure S5 Visualization of mitochondrial genomes sequence alignment between *Marasmius crinis-equi* (GHA76) and *Marasmius tenuissimus* (GHA63). The mVISTA program (http://genome.lbl.gov/vista/mvista/submit.shtml) was used to compare the complete mitochondrial genomes of *M. crinis-equi* with of *M. tenuissimus*, taking the annotation of the mitochondrial genome of *M. crinis-equi* as a reference. Default parameters were utilized to align the genomes in Shuffle-LAGAN mode and a sequence conservation profile was visualized in an mVISTA plot (Frazer et al., 2004).

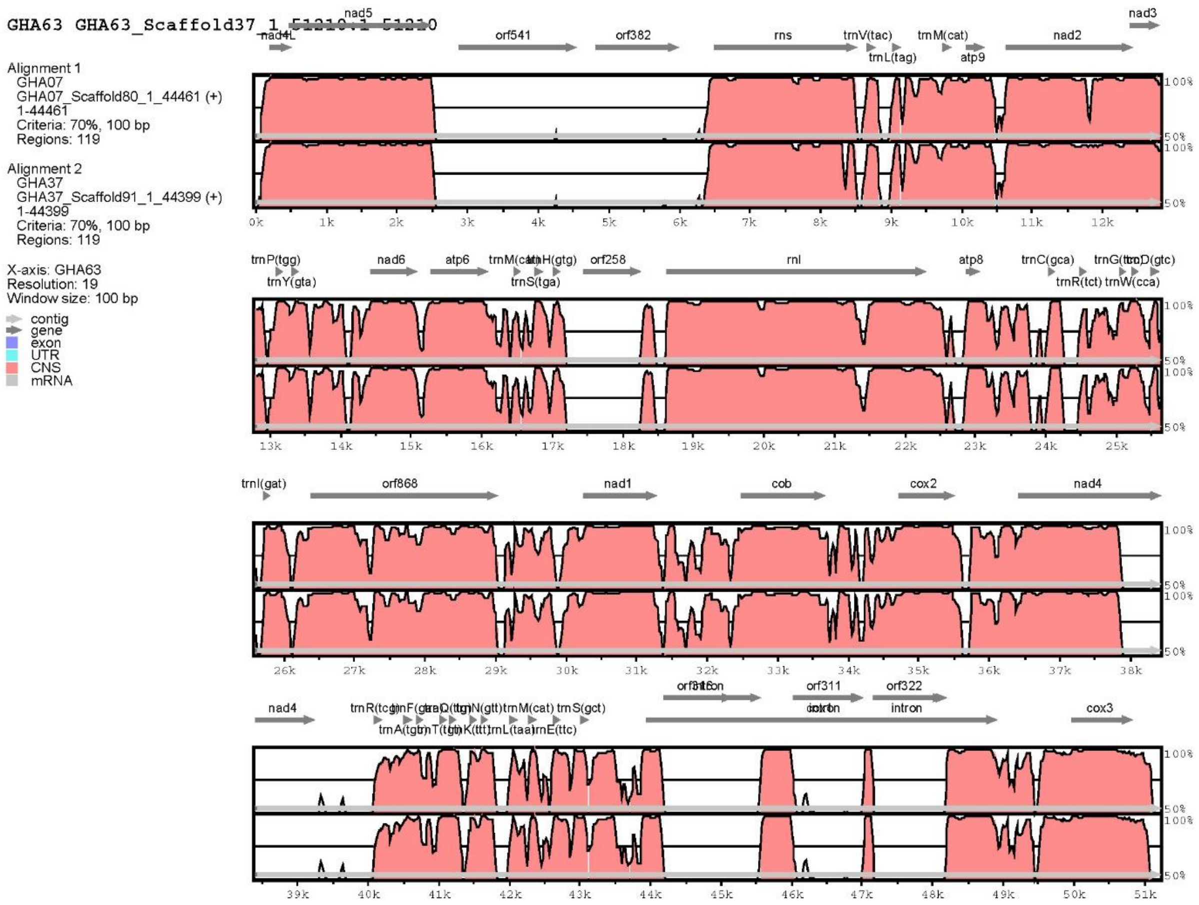

Supplementary Figure S6 Visualization of mitochondrial genomes sequence alignment between *Marasmius tenuissimus* isolate GHA63, GHA07 and GHA37. The mVISTA program (http://genome.lbl.gov/vista/mvista/submit.shtml) was used to compare the complete mitochondrial genomes of *M. tenuissimus* isolate GHA63 with GHA07 and GHA37 taking the annotation of the mitochondrial genome of isolate GHA63 as a reference. Default parameters were utilized to align the genomes in Shuffle-LAGAN mode and a sequence conservation profile was visualized in an mVISTA plot (Frazer et al., 2004).

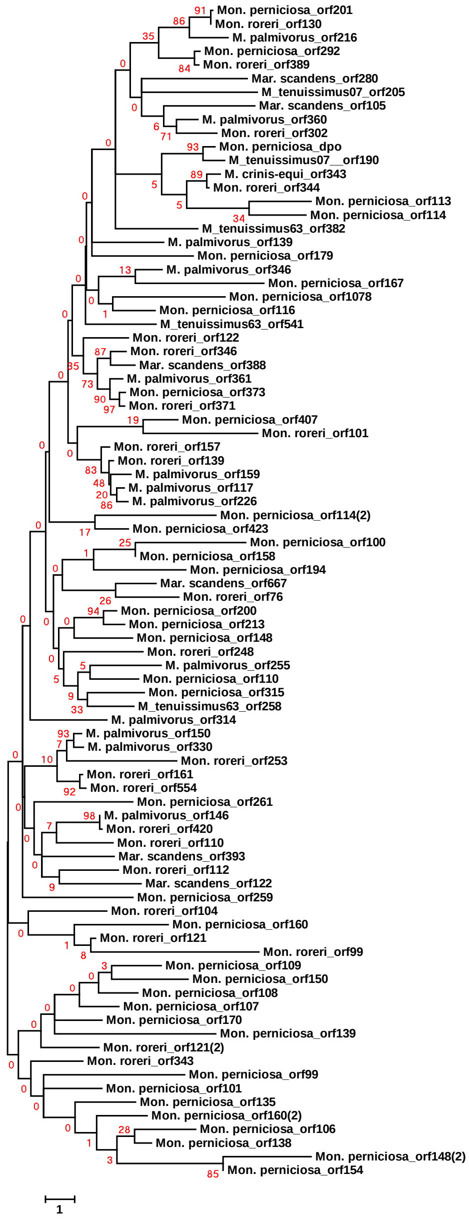

Supplemental Figure S7 Molecular phylogenetic analysis of the external unidentified ORFs (uORF) of four cacao thread blight associated pathogens from Ghana (*M. tenuissimus*, *M. palmivorus*, *M. crinis-equi*, *Mar. scandens*) and the Western hemisphere frosty pod rot (*Mon. roreri*) and witches' broom (*Mon. perniciosa*) pathogens of cacao. The analysis was based on amino-acid sequences of uORF from each isolate with 1282 distinct alignment positions and 1000 rapid bootstrap inferences. Sequences were combined and aligned using ClustalW2 tool under default setting and the phylogenetic tree was reconstructed using the Maximum Likelihood method. The tree is drawn to scale, with branch lengths measured in the number of substitutions per site. Analyses were conducted in MEGA6 (Tamura et al. 2013).

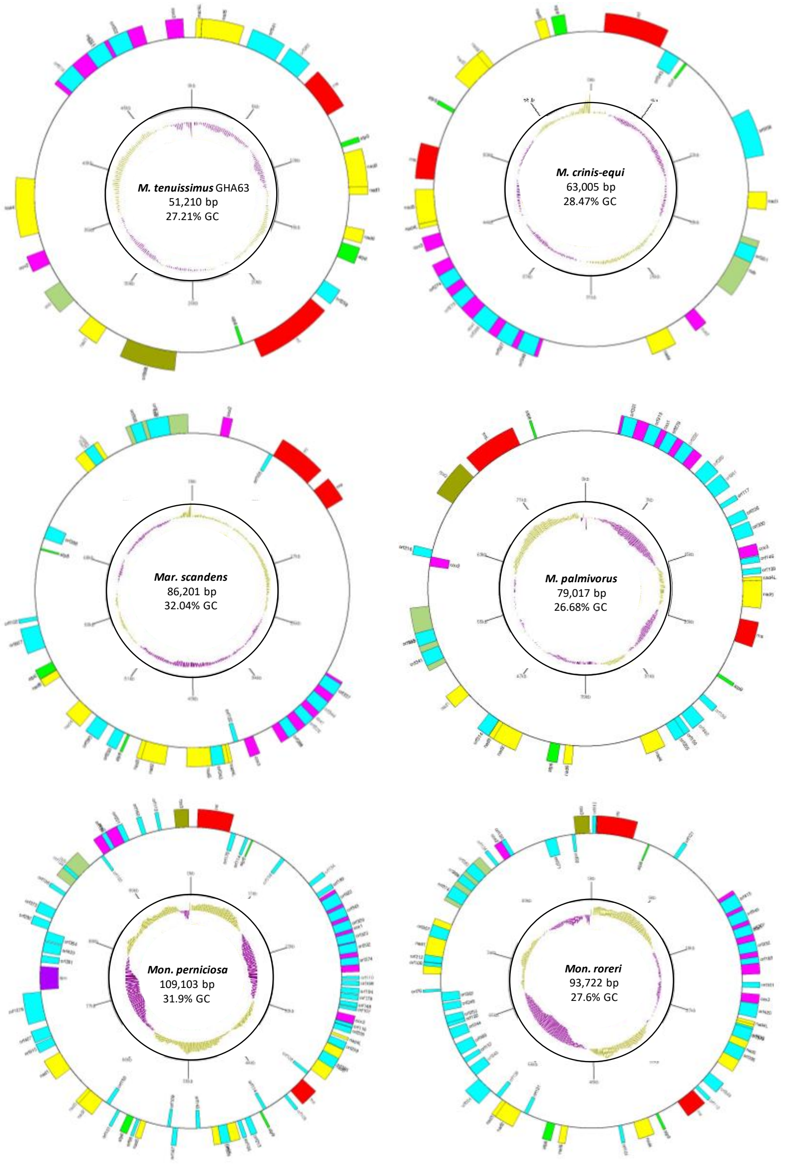

Supplementary Figure S8 The mitochondrial genome structure and GC content of four cacao thread blight associated pathogens from Ghana and the Western hemisphere frosty pod rot (*Mon. roreri*) and witches' broom (*Mon. perniciosa*) pathogens of cacao. Mitochondrial gene annotation was performed with Mfannot and the physical map was generated using GenomeVx. The GCplots (inner most circle) were created using DNAplotter (Release 18.1.0) with a window size of 10,000, and step size of 200. The pink represents below average and the green represents above average GC content.

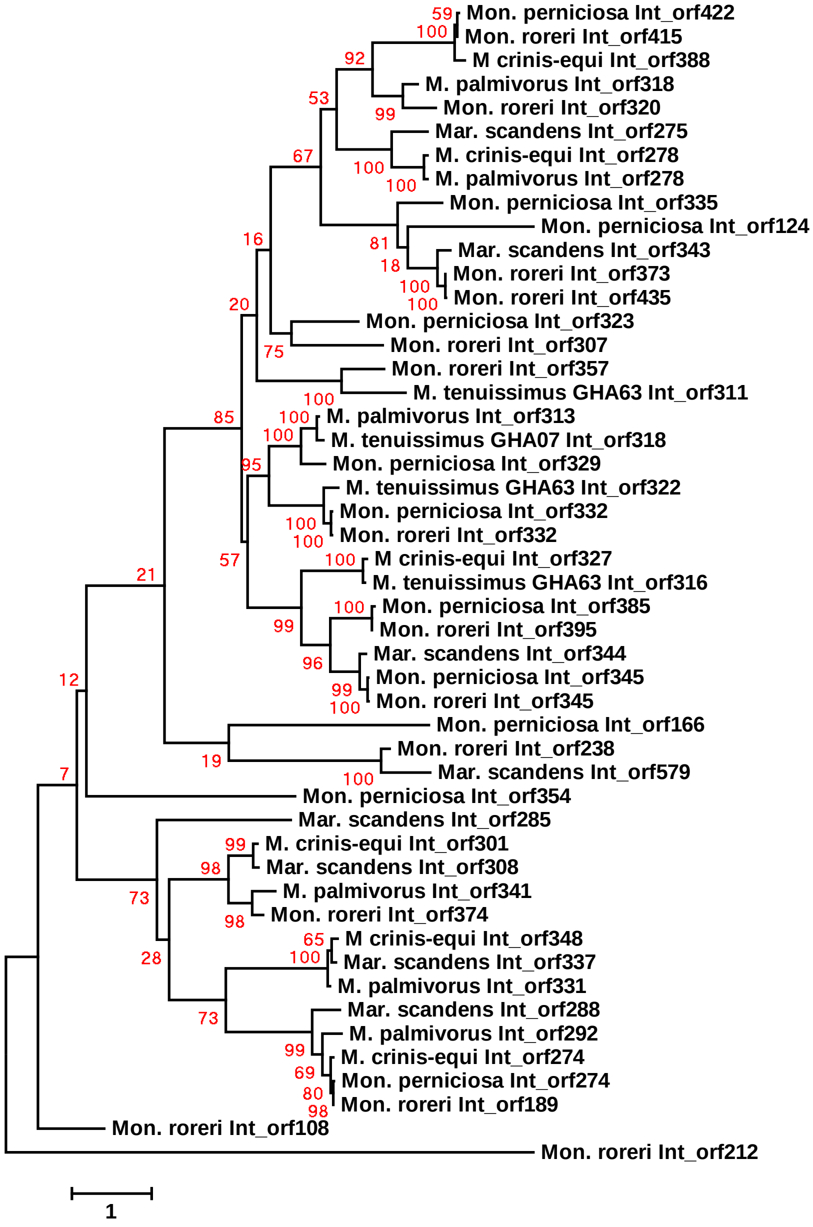

Supplementary Figure S9 Molecular phylogenetic analysis of the internal ORF's of four cacao thread blight associated pathogens from Ghana (*M. tenuissimus*, *M. palmivorus*, *M. crinis-equi*, *Mar. scandens*) and the Western hemisphere frosty pod rot (*Mon. roreri*) and witches' broom (*Mon. perniciosa*) pathogens of cacao. The analysis was based on amino-acid sequences of ORF's from each isolate with 700 distinct alignment positions and 1000 rapid bootstrap inferences. Sequences were combined and aligned using ClustalW2 tool under default setting and the phylogenetic tree was reconstructed using the Maximum Likelihood method. The tree is drawn to scale, with branch lengths measured in the number of substitutions per site. Analyses were conducted in MEGA6 (Tamura et al. 2013).
